# Efficient development of stable and highly functionalised peptides targeting the CK2α/CK2β protein–protein interaction†
†Electronic supplementary information (ESI) available. See DOI: 10.1039/c9sc00798a


**DOI:** 10.1039/c9sc00798a

**Published:** 2019-04-12

**Authors:** Jessica Iegre, Paul Brear, David J. Baker, Yaw Sing Tan, Eleanor L. Atkinson, Hannah F. Sore, Daniel H. O' Donovan, Chandra S. Verma, Marko Hyvönen, David R. Spring

**Affiliations:** a Department of Chemistry , University of Cambridge , Lensfield Road , CB2 1EW , Cambridge , UK . Email: spring@ch.cam.ac.uk; b Department of Biochemistry , University of Cambridge , Tennis Court Road , CB2 1GA , Cambridge , UK . Email: mh256@cam.ac.uk; c Discovery Sciences , IMED Biotech Unit , AstraZeneca , Cambridge , UK; d Bioinformatics Institute , Agency for Science, Technology and Research (A*STAR) , 30 Biopolis Street, #07-01 Matrix , Singapore 138671; e Oncology, IMED Biotech Unit , AstraZeneca , Cambridge , UK; f Department of Biological Sciences , National University of Singapore , 14 Science Drive 4 , Singapore 117543; g School of Biological Sciences , Nanyang Technological University , 60 Nanyang Drive , Singapore 637551

## Abstract

The discovery of new Protein–Protein Interaction (PPI) modulators is currently limited by the difficulties associated with the design and synthesis of selective small molecule inhibitors. Peptides are a potential solution for disrupting PPIs; however, they typically suffer from poor stability *in vivo* and limited tissue penetration hampering their wide spread use as new chemical biology tools and potential therapeutics. In this work, a combination of CuAAC chemistry, molecular modelling, X-ray crystallography, and biological validation allowed us to develop highly functionalised peptide PPI inhibitors of the protein CK2. The lead peptide, **CAM7117**, prevents the formation of the holoenzyme assembly *in vitro*, slows down proliferation, induces apoptosis in cancer cells and is stable in human serum. **CAM7117** could aid the development of novel CK2 inhibitors acting at the interface and help to fully understand the intracellular pathways involving CK2. Importantly, the approach adopted herein could be applied to many PPI targets and has the potential to ease the study of PPIs by efficiently providing access to functionalised peptides.

## Abstract 2

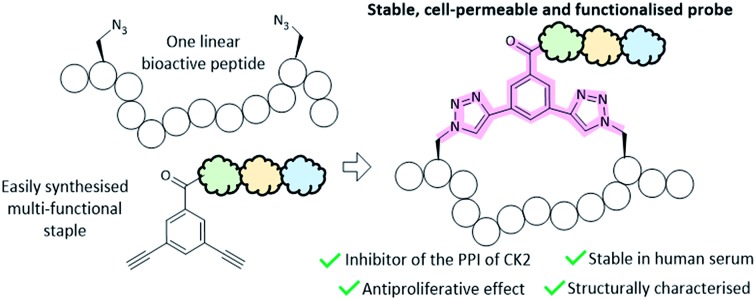
This work describes the efficient development of functionalised, cell-permeable, and stable peptide inhibitors of the protein–protein interaction of CK2.

## Introduction

Proteins are an essential component of the cell. They exert physio-pathological functions in response to external and internal messengers in a highly regulated and selective manner. Many of these functions are made possible by a complicated network of protein–protein interactions (PPIs). Targeting of PPIs is seen as an attractive therapeutic strategy considering the large number of PPIs involved in pathological mechanisms. In addition, targeting of PPIs provides orthogonality with respect to conventional therapeutics, which are typically targeted against existing binding sites for small molecules. Therefore, inhibition of PPIs may result in the development of safer drugs.^1^ PPIs are characterised by shallow surfaces which make targeting them with conventional small molecules (<500 Da), whilst possible,^2^–^7^ a non-trivial and lengthy process. Synthetic peptides, on the other hand, provide a valuable alternative to small molecules: their structural properties make them amenable to mimic portions of the native proteins resulting in favorable interactions with the large and shallow interfaces of PPIs.^8^ In addition, when structural information pertaining to the PPI of interest is known, potent and selective peptides can be rapidly designed based upon the sequence of targeted motifs of the native proteins.^9^

However, the poor pharmacokinetic (PK) properties of synthetic peptides in the body limit their use and interest to the pharmaceutical industry.^10^ Many strategies have been adopted to overcome the PK limitations of peptides; yet, very few of them are set up to provide peptides that are highly functionalised and cell-permeable in an efficient manner.^11^–^15^ Taking into account the potency and selectivity of peptides, methodologies that introduce functionalities to make them simultaneously stable and cell-permeable would yield chemical probes of invaluable importance.^16^ To this end, we have developed a two-component (2C) copper catalyzed azido alkyne (CuAAC) peptide stapling (PS) methodology that allows the development of highly functionalised, potent, and selective peptides targeting intracellular PPIs.^17^ In 2C-CuAAC-PS only a limited number of peptides are synthesised and their design is based upon the structural information available for the PPI of interest.^18^ Subsequently, peptide stapling is carried out by using amino acids with azido side chains and a di-alkyne linker as a constraint. One of the advantages of this methodology is that the linker can be modified and functionalised to improve the *in vitro* pharmacological properties independently from the peptide sequence: the result is a more efficient optimisation of the peptides. Biophysical and cellular assays are successively used to assess the peptides synthesised. Importantly, the 2C-CuAAC-PS chemistry proved to be compatible with all the natural amino acids, led to enhanced binding affinities for both helical and non-helical peptides and improved the overall *in vitro* pharmacological properties of the peptides, including stability to proteases.^19^–^21^

Herein, we have applied this robust approach to efficiently develop the first stable and highly functionalised conformationally-constrained peptide acting on the PPI of CK2.

**Fig. 1:**
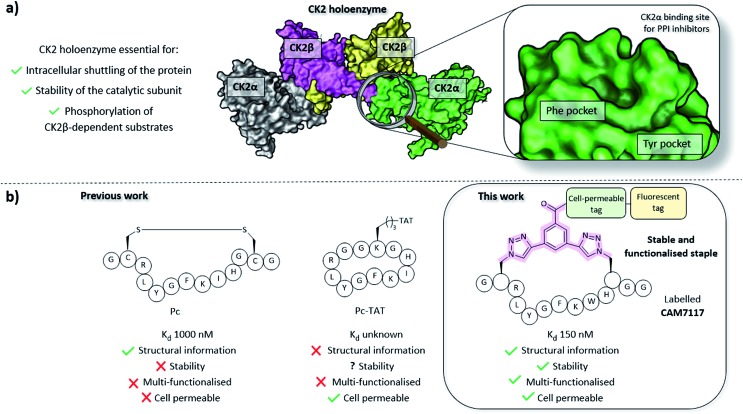
(a) Importance of the holoenzyme to the functions of CK2 (PDB: 1JWH). The catalytic α subunits are shown in grey and green, the regulatory β subunits in yellow and pink. The binding site on CK2α for inhibitors of the PPI is shown on the right. (b) A comparison of the peptides developed prior to this work (Pc and TAT-Pc)^26^,^28^,^34^ and the lead peptide **CAM7117** developed in this work.

CK2 is a protein kinase overexpressed in cancer cells and a validated oncology target; CX4945, a traditional small molecule ATP-binding site inhibitor of CK2, is currently undergoing clinical studies.^22^ However, CX4945 targets the ATP-binding site, which is well conserved among the kinome. More recently, there have been increasing efforts to develop non-ATP competitive inhibitors of CK2 to reduce the off-target effects of competitive ligands.^23^–^25^ Among the strategies designed to target CK2 outside its orthosteric binding site, is the inhibition of the PPI between the α and the β subunits.^26^–^29^ Disruption of the holoenzyme assembly affects the function of CK2 by preventing phosphorylation of β-dependent substrates, the shuttling of the protein between different intracellular compartments, and by reducing the stability of the catalytic α subunit (Fig. 1).^30^–^33^ With the exception of the Phe pocket, the CK2α/β interface is a shallow and hydrophobic surface; consequently, peptides are an ideal class of molecule to target this PPI. To this end, two cyclic peptides have been developed. However, one of these, Pc,^26^,^28^ is a disulfide-linked cyclic peptide that lacks cell permeability and stability in the reducing intracellular environment, and the other, TAT-Pc,^34^ has not been assessed structurally or for stability in physiologic fluids (Fig. 1). Therefore, a stable chemical probe that could be used *in vitro* and *in vivo* to study the interface of the important protein CK2 is still required.

Starting from the sequences of CK2β and Pc, we investigated alternative ways of constraining the peptide into its bioactive conformation using a stable linkage compatible with the 2C-CuAAC-PS chemistry. At a later stage, X-ray crystallography guided our investigation on sequence variation to increase the binding affinity of the peptide for CK2α. The most promising peptide was easily modified into a fluorescent, cell-permeable probe *via* a novel highly functionalised constraint that allowed us to study the peptide's activity in cancer cells.

The peptide developed in this work is the first stable, cell permeable macrocyclic peptide that disrupts the CK2α/β PPI *in vitro* and leads to cancer cell death and arrest of the cell cycle; as such, it will serve as a useful chemical probe in oncology. Furthermore, the structure of the peptide in complex with CK2α will act as a valuable starting point to develop novel CK2 inhibitors.

## Results and discussion

In order to design stable peptides targeting the CK2α/β interaction, we used a rational-design approach based on the valuable crystal structures of CK2β and the disulfide bridged Pc peptide.^34^

Disulfide bridges are unstable under reducing environments; therefore, our aim was to replace the labile disulfide group with a stable constraint. To this end, the 2C-CuAAC macrocyclisation technique was chosen for its validated ability to constrain peptides in their binding conformation, simultaneously enhance the stability against proteolytic cleavage, introduce functionalities (cell-penetrating peptide (CPP), fluorescent dyes, biotin, and PEG and other tags), and improve the poor stability in physiological fluids and cell-penetration in a combinatorial manner.^19^,^20^,^35^–^38^

### Rational design of conformationally constrained peptides mimicking CK2β

**Fig. 2:**
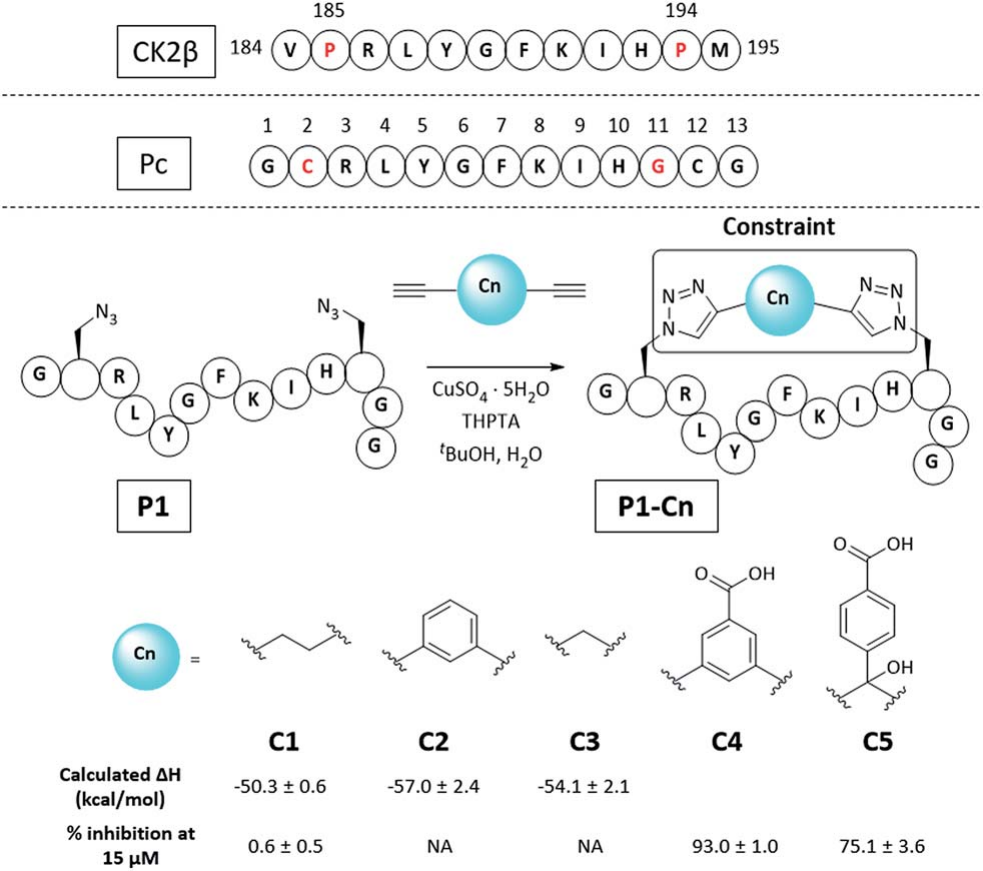
Sequence of the portion of CK2β binding to CK2α, Pc peptide and structure of the constrained peptides reported in this work with calculated enthalpic values of binding and percentage of CK2β-like probe displacement. NA = not measured. A detailed table with the structures of all the peptides presented in this work is provided in the ESI (Table S2†). Pc, **P1**, and **P1-Cn** (where *n* = 1–5) peptides feature an amide at the C-terminus and an acetyl cap at the N-terminus. All the amino acids are the l isomers.

Molecular modelling identified Cys2 and Gly11 of Pc,^34^ corresponding to P185 and P194 of CK2β, as suitable residues to staple: they make negligible contributions to the binding and are positioned at a suitable distance from each other to accommodate a 2C-CuAAC staple (ESI, Fig. S1†). To cyclise the peptide, azido amino acids bearing one-carbon-atom side chains (Fmoc-Aza-OH) were used in combination with aliphatic linkers of different lengths as proposed by molecular modelling (Fig. 2 and ESI Table S1†). The ability of the synthesised macrocycles to disrupt the α/β PPI of CK2 was then tested in a preliminary fluorescent polarisation assay (FP) (Fig. 2).

Peptides constrained with linkers **C2** and **C3** proved to be insoluble under the assay conditions. To overcome this limitation, it was decided to incorporate a functional handle on the constraint to simultaneously improve the solubility and allow for future functionalisation of the peptides. Peptide **P1-C4** was the most effective macrocyclised peptide at displacing the FP probe from the CK2α subunit (93 ± 1% inhibition at 15 μM). This suggested that constraint **C4** was optimal at constraining the peptide in its binding conformation. The binding affinity of **P1-C4** and **P1-C5** for the CK2α subunit was measured *via* isothermal titration calorimetry (ITC). **P1-C4** showed the highest affinity with a *K*_d_ of 460 nM, showing a modest increase compared to Pc (1000 nM, ESI Table S3†).

**Fig. 3:**
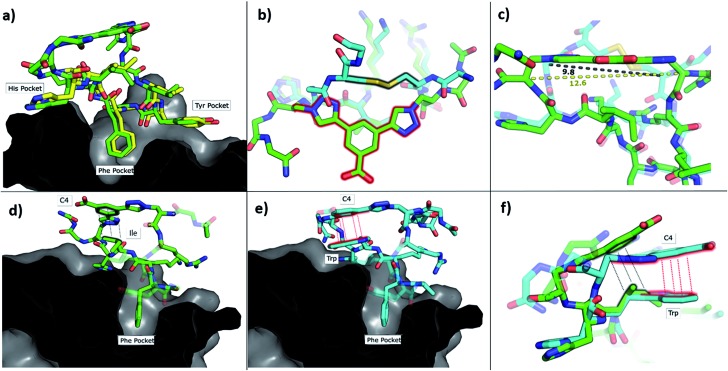
Crystallographic structures of conformationally constrained peptides. (a) **P1-C4** (green, PDB: ; 6Q38) and residues 186-193 of CK2β (yellow, PDB: ; 4NH1 (ref. 44)) in complex with CK2α. The image is shown as a cross-section of the interface site. (b) A comparison of the binding mode of the two different linkers in Pc (cyan, PDB: ; 4IB5 (ref. 34)) and **P1-C4** (green). (c) Difference in the distance between the α-carbons of the amino acids involved in the constraint in Pc (cyan) and **P1-C4** (green). (d) Crystallographic structure of **P1-C4** (green) binding at the interface site. The image is shown as a cross-section of the interface site. (e) Crystallographic structure of **P2-C4** (cyan, PDB: ; 6Q4Q) binding at the interface site. Stacking of the Trp residue with the constraint is highlighted in red. The image is shown as a cross-section of the interface site. (f) Comparison of the binding mode of **P1-C4** (green) and **P2-C4** (cyan) bound to the interface site.

To understand how **P1-C4** was able to bind CK2α, we determined its crystal structure in complex with CK2α (PDB: ; 6Q38). The crystal structure showed the peptide binding in a conformation that overlays well with CK2β and Pc (Fig. 3a–c). The backbone residues of **P1-C4** are all slightly shifted compared to Pc; however, these differences are greatest closer to the constraint that holds the two ends of the peptide further apart than the disulfide linker does; consequently, the terminal residues adopt entirely different conformations (Fig. 3b and c).

The X-ray crystal structure of **P1-C4** (Fig. 3d) shows Ile9 oriented in the right direction to be replaced by an amino acid capable of π–π stacking with the benzene ring of the **C4** constraint. Similarly, chlorobenzene probes in ligand-mapping MD simulations^39^,^40^ of **P1-C4** indicated a region of high occupancy by the aromatic carbon atoms of chlorobenzene around Ile9 of the peptide (ESI Table S1†). Therefore, the sequence of **P1** was modified to replace Ile9 with a larger, non-polar Trp to create peptide **P2** (Ac-GXRLYGFKWHXGG-NH_2_ where X = Fmoc-Aza-OH). The resulting peptide was macrocyclised using **C4** as a linker to afford **P2-C4**.

**Fig. 4:**
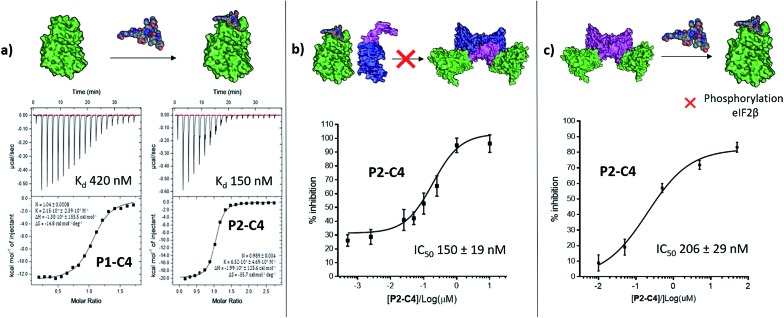
*In vitro* assessment of **P1-C4** and **P2-C4**. (a) ITC binding curves of **P1-C4** and **P2-C4**. A schematic representation of the peptide association with CK2α (green) is shown on the top of the ITC curves. (b) Ability of **P2-C4** to cause inhibition of the association of the CK2α/CK2β-like holoenzyme using CK2α (green) and CK2β-RAD (pink and blue). (c) Ability of **P2-C4** to cause inhibition of the catalytic activity of CK2α (green) towards a CK2β-dependent substrate eIF2β by **P2-C4**. Starting CK2 holoenzyme is shown in green and pink; peptide is shown as spheres.

The binding affinity of the **P2-C4** peptide was found to be enhanced (*K*_d_ 150 nM) compared to both **P1-C4** (*K*_d_ 460 nM) and Pc peptide (*K*_d_ 1000 nM) (Fig. 4a and ESI Table S3†). The constrained **P2-C4** peptide showed also a 300-fold improvement compared to the linear variant **P2** (*K*_d_ 44 μM). The crystal structure of **P2-C4** bound to CK2α (PDB: ; 6Q4Q, Fig. 3e and f) shows the π–π stacking between the Trp and the phenyl ring of the constraint. It is likely that this π–π stacking is the main factor leading to the higher affinity of **P2-C4** as no other significant interactions were observed (ESI Fig. S2†). Therefore, the increased binding affinity of **P2-C4** for CK2α may be explained with a reduced entropic penalty upon binding due to the rigidifying interaction occurring between the constraint and the Trp residue.^41^–^43^

### Inhibition of the CK2α/β protein–protein interaction

We then wanted to investigate whether the peptides would be able to inhibit the binding of the β subunit to the α subunit. To this end, the binding affinity of the regulatory β subunit for the catalytic domain was determined *via* ITC (*K*_d_ 9 nM). This was then repeated in the presence of 100 μM **P1-C4** and **P2-C4** (ESI, Table S4†). This preliminary assay showed that no binding of CK2β to CK2α was detected in the presence of either of the peptides: this result confirms that the peptides bind at the interface and prevent CK2β binding despite its high binding affinity for CK2α. As an orthogonal proof of inhibition, we used competition Bio-Layer Interferometry (BLI) experiments to demonstrate that **P2-C4** was able to prevent the formation of the CK2α/CKβ-RAD‡
‡CK2α-interacting loop of CK2β (GGLKRLYGFKIHPMAYQLQLKGG) displayed on the biotinylated globular ATPase domain of recombinase RadA.^48^ complex with an IC_50_ of 150 ± 19 nM (Fig. 4b). As one of the key roles of CK2β is to recruit substrates to the kinase, the effect of PPI inhibition on substrate phosphorylation was studied using CK2β dependent and independent substrates. It was shown that **P2-C4** was able to inhibit the phosphorylation of a CK2β-dependent substrate – the transcription factor eIF2β – with an IC_50_ of 206 ± 29 nM (Fig. 4c). It should be noted that the kinase assay was performed using the pre-formed CK2α/β complex as the ability of CK2α to phosphorylate eIF2β requires the presence of the CK2β subunit. The assay indicates that **P2-C4** can disrupt, in a dose-dependent manner, the CK2 holoenzyme thereby reducing the ability of CK2α to phosphorylate CK2β-dependent substrates. As expected, **P2-C4** did not affect the phosphorylation of a β-independent substrate peptide (RRRADDSDDDD) meaning that binding at the interface site does not displace the ATP or significantly alter the kinase activity allosterically (ESI Fig. S3†).

### Development of a multi-functional constraint

**Fig. 5:**
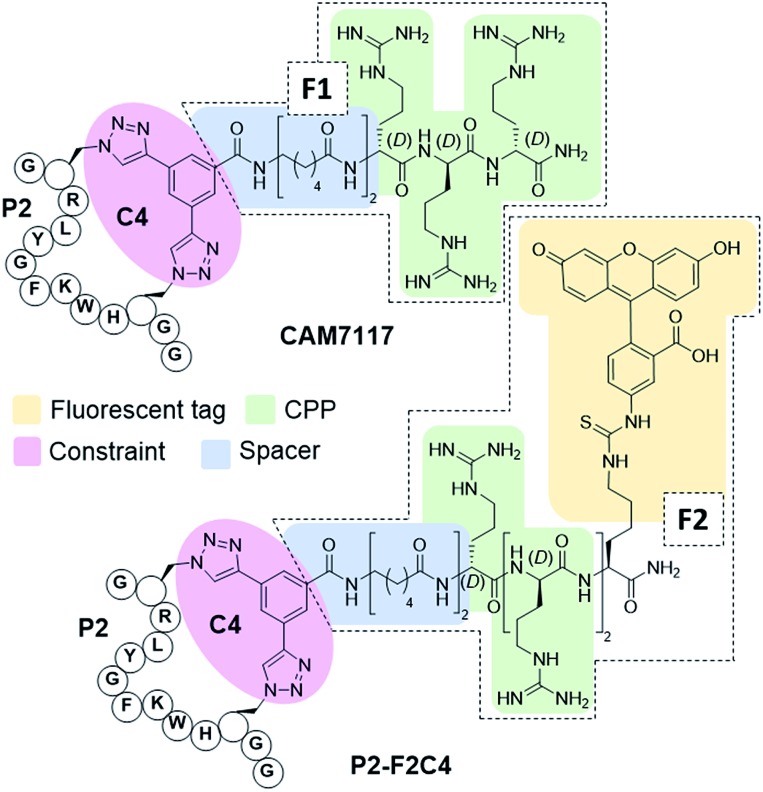
Structures of the multi-functionalised peptides. The peptides are constrained with a multifunctional linker containing a linkage core that locks the peptide in its binding conformation and enhances stability to proteases (pink), a spacer to avoid steric clashes (blue), a protease-resistant poly(d)arginine tag to gain cellular permeability (green) and a fluorescent tag to monitor intracellular localisation (yellow).

To overcome the limitations of Pc, the main goal of this work was to efficiently develop chemical probes for the CK2 PPI that could be used in cells and ultimately *in vivo*. Therefore, cell-permeability and stability in serum were required. Peptide macrocyclisation has been described on several occasions as a powerful technique to enhance the stability of the peptides to proteases.^20^,^21^,^45^ Although the ability of peptide macrocyclisation alone to enhance cell-permeability is highly debated, cell-penetrating peptides (CPPs) are well-established and are often added to the cyclised peptides to gain cytosolic entry.^46^,^47^ Preliminary cellular uptake experiments carried out with FITC-labelled peptide showed that the peptide was not able to permeate the cell membrane of osteosarcoma cancer cells (U2OS) and thus, functionalisation was needed. Considering that both cell-penetrating motifs and fluorescent tags were necessary for the cellular assays, we decided to develop a novel multi-functional constraint that would simultaneously: constrain the peptide in its binding conformation, enhance the stability to proteases, provide cell-permeability to the CK2 peptide, and act as a fluorophore (**F2C4**, Fig. 5). In addition, we wanted to develop functionalised constraints that could be synthesised in an automated manner using Fmoc-SPPS so to be accessible by the wider scientific community. Exploiting one of the advantages of 2C-PS, linker **C4** was elaborated independently from the CK2 peptide (ESI Scheme S1†). The benzoic acid derivative linker **C4** (pink in Fig. 5) was attached to a CPP *via* a spacer (blue in Fig. 5) to avoid steric clashes with the CK2 peptide and the CK2α domain, generating the full multi-functional linker **F1C4**. Previously, we have reported the use of an (l)-arginine tripeptide as an effective CPP to carry peptide cargos into cells.^20^,^36^ We decided to use (d)-arginine instead to confer cell-permeability to the CK2 peptide and simultaneously provide a proteolysis resistant alternative. The CPP was in turn attached to the fluorescent tag FITC *via* an orthogonally protected Lys to monitor the peptide entrance into the cells (full multi-functional linker **F2C4**, Fig. 5). A fluorescent constraint without the CPP motif (**F3C4**) was also synthesised. The functionalised linker **F1C4** was then reacted with **P2** to obtain **CAM7117** (Fig. 5). Importantly, **CAM7117** displayed significant stability in human serum (47% intact peptide after 24 hours incubation, Fig. S4†) highlighting how the covalent constraint **F1C4** provided a peptide that is stable under physiological fluids.

### Activity of **CAM7117** in cancer cells

**Fig. 6:**
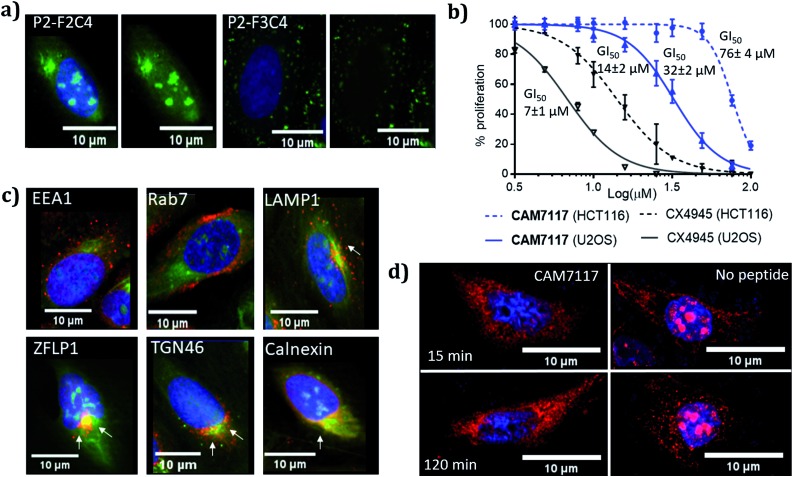
Biological evaluation of **CAM7117** and its analogues in cancer cells. (a) Cellular uptake in U2OS cell line for **P2-F2C4** and **P2-F3C4**. Cell nuclei are stained with Hoechst 33342 stain (blue). (b) GI_50_ curves of the antiproliferative activity of **CAM7117** (solid and dashed blue lines) and CX4945 (solid and dashed black line) in U2OS and HCT116 cells. (c) Immunofluorescence experiments in the presence of FITC-labelled peptide **P2-F2C4** (green), stained with antibodies against markers of different organelles (all in red): EEA1 (early endosome), Rab7 (late endosome), LAMP1 (lysosomes), ZFLP1 (*cis* Golgi), TGN46 (*trans* Golgi) and calnexin (endoplasmic reticulum). Details of antibodies are found in Table S11.† Nuclei are stained with Hoechst 33342 (blue). (d) Change in intracellular localisation of CK2β (red) following treatment with **CAM7117** (30 μM) for 15 and 120 minutes. Nuclei are stained with Hoechst 33342 (blue).

**CAM7117** was successfully internalised by the cancer cells as observed by confocal microscopy of its FITC-labelled analogue, **P2-F2C4** (Fig. 6a). Once internalised, the peptide was able to inhibit human osteosarcoma (U2OS) cell growth with a GI_50_ of 32 ± 2 μM after 4 day incubation, and induced apoptosis after just 4 hours (ESI Fig. S5–S7†). A marginally reduced biological effect was observed for both **CAM7117** and the clinical candidate CX4945 when human colorectal cancer cells (HCT116) were used (Fig. 6b). No biological effects were observed when the functionalised constraint only (**F1C4**) or the negative control peptide (**P3-F1C4**) were used. The negative control **P3-F1C4** features an F7W mutation in the sequence and did not show binding to CK2α by ITC (ESI Table S3, Fig. S5–S7†).

Although specific biological activity was observed in cancer cells, the drop-off between the enzymatic and cellular assays was higher than expected. Therefore, we investigated the effect of intracellular localisation of the FITC-labelled **CAM7117**, and imaging experiments were carried out to look at co-localisation with several organelle markers. We were unable to detect any co-localisation with endosomal markers (Fig. 6c, ESI Fig. S8 and S9†), but partial co-localisation was detected with lysosomal marker, and a significant amount of the peptide was found to localise to the same place as the Golgi and endoplasmic reticulum (ER) markers (Fig. 6c, ESI Fig. S8 and S9†). A significant proportion of the peptide was able to diffuse to the cytosol and localised mainly in the nucleus (Fig. 6c).

Therefore, the drop-off in cellular activity may be attributed to trapping of the peptide in the Golgi/ER. Moreover, the activity drop-off in cells could also be due to the mechanism of inhibition: unlike the inhibition of the α catalytic domain (such as CX4945), displacing the β subunit does not inhibit the phosphorylation of all CK2 substrates.

Further imaging experiments using U2OS cells were carried out to evaluate the effect of **CAM7117** in subcellular localisation of the CK2 subunits.

Already after 15 minute incubation, little or no fluorescence associated with the CK2β antibody was found in the nucleus; on the contrary, untreated cells showed significant punctate staining nuclear accumulation of the CK2β (Fig. 6d). This evidence suggests that **CAM7117** could engage with CK2α and compete with CK2β in a cellular context.

## Conclusions

In the present study, we have showcased an approach to efficiently develop peptides that are highly functionalised, cell-permeable and stable in serum. In particular, using the 2C-CuAAC-PS methodology, we have developed conformationally constrained peptides that act as inhibitors of the CK2 α/β PPI *in vitro* and in cancer cells. The lead peptide, **CAM7117**, presents an enhanced binding affinity for CK2α with respect to the previously developed Pc and, most importantly, is stable under conditions mimicking physiological fluids. Owing to the lack of intrinsic cell-permeability of the peptides, we developed an easily-synthesised multi-functional constraint that allowed us to investigate the intracellular activity of **CAM7117**, which arrests cancer cell proliferation and induces apoptosis in a dose-dependent manner. To the best of our knowledge, this study describes the development of the first inhibitory peptide of the CK2α/β PPI that is: stable in serum, cell permeable, active in cells, able to engage the target and structurally characterised. Such a peptide would act as a chemical probe that enables the study of the CK2 PPI using endogenous levels of proteins and could, therefore, be used to elucidate CK2 dependent mechanisms leading to cancer progression.

Remarkably, the multi-functional constraint developed in this work could be used to lock other peptides into their binding conformation and simultaneously functionalise them. Therefore, the strategy adopted herein could, provide a more universal approach to develop modulators of PPIs for many targets where linear sequence epitope provides the majority of the binding energy. Moreover, considering that the peptide addition to either cells or organisms can be done with rigorous temporal and quantitative control, these probes are extremely powerful tools for validating PPIs in drug discovery and dissecting biological processes.

## Conflicts of interest

There are no conflicts to declare.

## Supplementary Material

Supplementary information
