# Supplementary material for: Efficient development of stable and highly functionalised peptides targeting the CK2α/CK2β protein–protein interaction
Source: Chem Sci. 2019 Apr 12;10(19):5056–63. doi: 10.1039/c9sc00798a (PMC6530537; doi:10.1039/c9sc00798a)
Supplement: Supplementary file 1 [file SC-010-C9SC00798A-s001.pdf]

Electronic Supplementary Material (ESI) for Chemical Science.
This journal is © The Royal Society of Chemistry 2019

Electronic Supporting Information

Efficient development of stable and highly functionalised peptides targeting the CK2α/CK2β protein-protein interaction

Jessica Iegre, Paul Brear, David J. Baker, Yaw Sing Tan, Eleanor L. Atkinson, Hannah F. Sore, Daniel H. O' Donovan, Chandra S. Verma, Marko Hyvönen,* David R. Spring*

Table of content

1.1. Figures, Tables and Schemes mentioned in the article 3
Figure S1 3
Table S1 3
Table S2 4
Table S3 6
Figure S2 8
Table S4 9
Figure S3 10
Scheme S1 10
Figure S4 11
Figure S5 11
Figure S6 12
Figure S7 13
Figure S8 13
Figure S9 14
1.2. Chemistry experimental 15
1.2.1. Experimental synthetic details 17
1.2.1.1. Small molecules 17
1.2.1.2. Peptides 29
1.3. Computational chemistry 36
1.3.1. Molecular dynamic simulations 36
1.3.1.1. Ligand-mapping MDs 36
1.3.2. Binding Free Energy Decomposition 37
1.3.3. Computational Alanine Scanning 37
1.3.4. Binding Free Energy Calculations 39
1.4. Biophysical experiments 39
1.4.1 Protein expression and purification 39
1.4.1.1. CK2α 39
1.4.1.2. CK2β 39
1.4.2. Fluorescent Polarisation 40
1.4.3. Isothermal titration calorimetry 40
1.4.3.1. Direct binding 40
1.4.3.2. Competition assay 41
1.4.4. Biolayer Interferometry (BLI) 41

1.4.5. X-ray crystallography 42
1.4.6. Kinase assay 45
1.5. Cellular biology 46
1.5.1. Serum stability test 46
1.5.2. Tissue culture 46
1.5.3. Proliferation assay 46
1.5.4. Cell viability assay 47
1.5.5. Confocal microscopy 47
1.6. References 49

1.1. Figures, Tables and Schemes mentioned in the main article

Figure S1

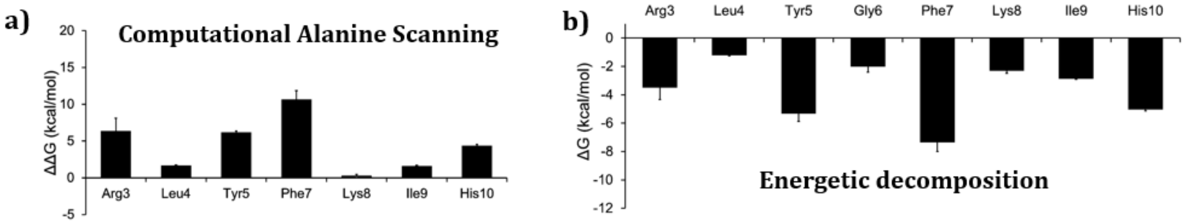

Figure S1 *- Results of the CAS (a) and Energetic decomposition (b) based on an ensemble of structures obtained from molecular dynamics (MD) simulations of the CK2α–Pc complex.*

Table S1

Table S1 *- Computed binding free energies (kcal/mol) of CK2 peptide ligands.*

Peptide	Sequence	ΔH	TΔS	ΔG
Pc	Ac-GCRLYGFKIHGCG-NH_2	-50.6 ± 1.7	-35.0 ± 1.7	-15.5 ± 0.3
short Pc	Ac-CRLYGFKIHGC-NH_2	-56.6 ± 1.2	-33.4 ± 1.4	-23.2 ± 2.0
**P0-C1***	Ac-X_{C1}RLYGFKIHX_{C1}-NH_2	-50.3 ± 0.6	-34.7 ± 1.1	-15.7 ± 1.4
**P1-C2**	Ac-GX_{C2}RLYGFKIHX_{C2}GG-NH_2	-57.0 ± 2.4	-40.6 ± 1.8	-16.4 ± 1.60
**P1-C3**	Ac-GX_{C3}RLYGFKIHX_{C3}GG-NH_2	-54.1 ± 2.1	-35.1 ± 1.6	-19.0 ± 3.2
**P2-C2**	Ac-GX_{C2}RLYGFKWHX_{C2}GG-NH_2	-59.7 ± 1.2	-37.8 ± 1.3	-21.8 ± 1.1

The shorter sequence **P0** was initially used as an attempt to remove the terminal Gly residues. However, cyclisation of the **P0** peptide was achieved when **C1** was as constraint only. No cyclisation was observed with the other constraints and therefore, the longer **P1** sequence was used.

Table S2

***Table S2** - Structure of the peptides presented in this study, Pc and CK2β*

Compound name	Sequence	Constraint
**CK2β (186-193)**	RLYGFKIH	NA
**Pc**	GCRLYGFKIHGCG	S-S

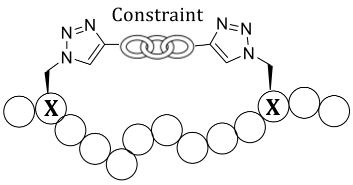

Compound name	Sequence	Constraint
**P0**	XRLYGFKIHX	NA
**P0-C1**	X_{C1}RLYGFKIHX_{C1}	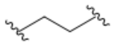
**P1**	GXRLYGFKIHXGG	NA
**P1-C2**	GX_{C2}RLYGFKIHX_{C2}GG	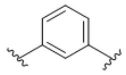
**P1-C3**	GX_{C3}RLYGFKIHX_{C3}GG	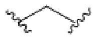
**P1-C4**	GX_{C4}RLYGFKIHX_{C4}GG	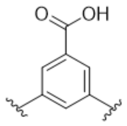
**P1-C5**	GX_{C5}RLYGFKIHX_{C5}GG	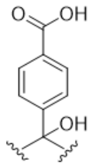
**P2**	GXRLYGFKWHXGG	NA
**P2-C4**	GX_{C4}RLYGFKWHX_{C4}GG	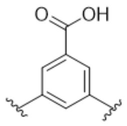

**P2-F2C4**	GX$_{F2C4}$RLYGFKWHX$_{F2C4}$GG	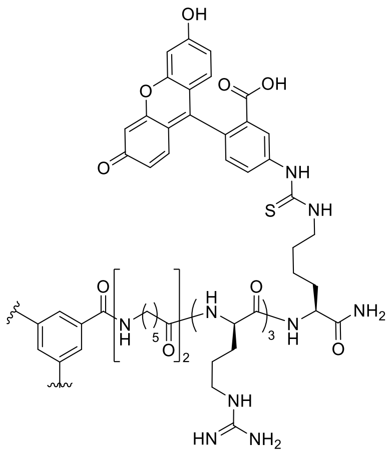
**CAM7117**	GX$_{F1C4}$RLYGFKWHX$_{F1C4}$GG	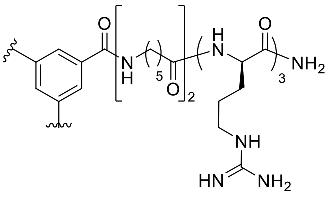
**P2-F3C4**	GX $_{F3C4}$RLYGFKWHX $_{F3C4}$GG	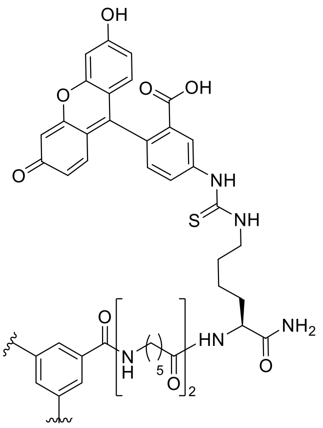
**P3**	GXRLYGWKIHXGG	NA
**P3-F2C4**	GX$_{F3C4}$RLYGWKIHX$_{F3C4}$GG	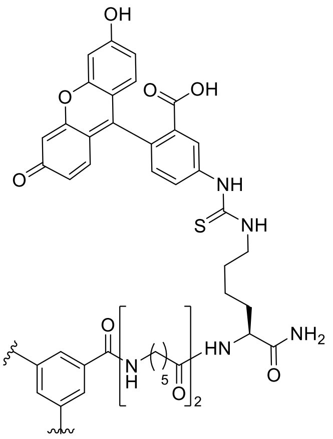

All the peptides present an amide at the C-terminus and the N-terminus is capped with an acetyl group

Table S3

***Table S3** – ITC binding curves of CK2 peptides to CK2α*

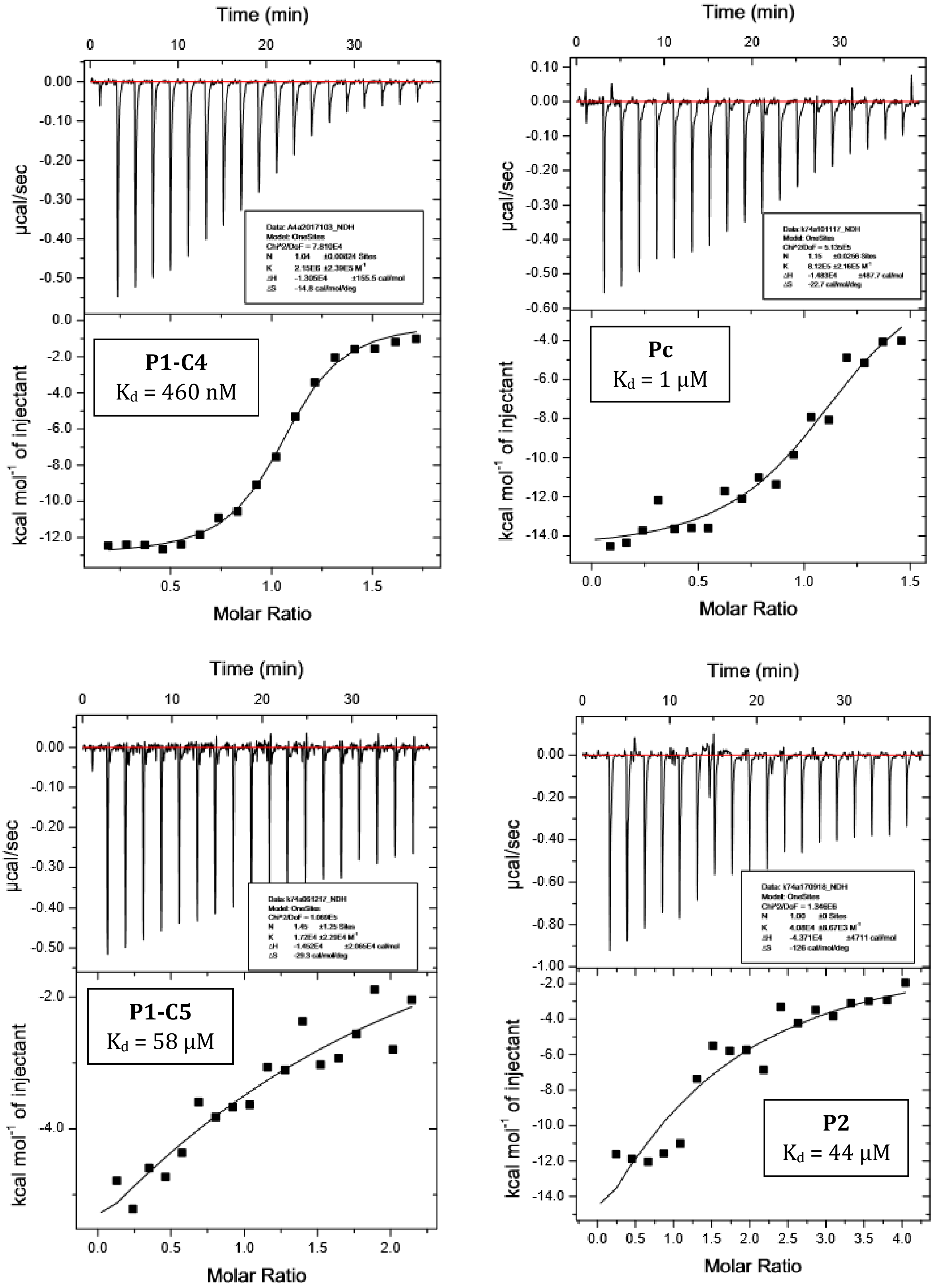

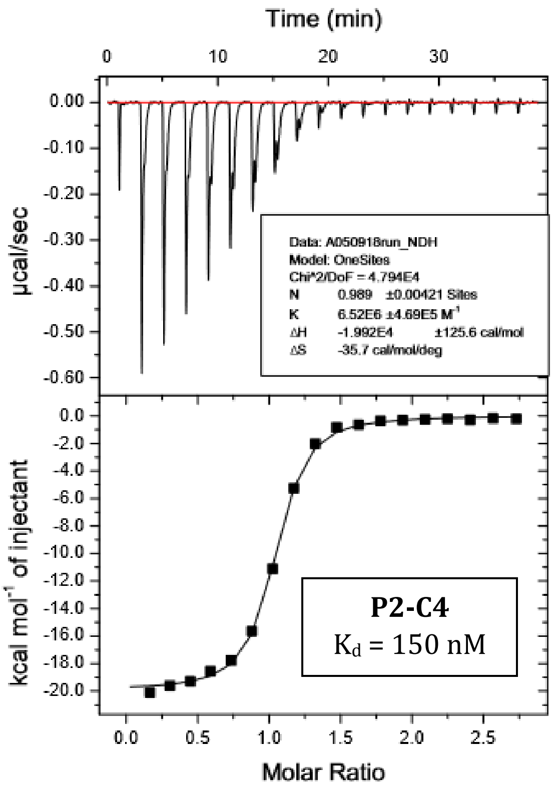
Time (min)
µcal/sec
Data: A050918run_NDH
Model: OneSites
Chi^2/DoF = 4.794E4
N 0.989 ±0.00421 Sites
K 6.52E6 ±4.69E5 M-1
ΔH -1.992E4 ±125.6 cal/mol
ΔS -35.7 cal/mol/deg
kcal mol-1 of injectant
P2-C4
Kd = 150 nM
Molar Ratio

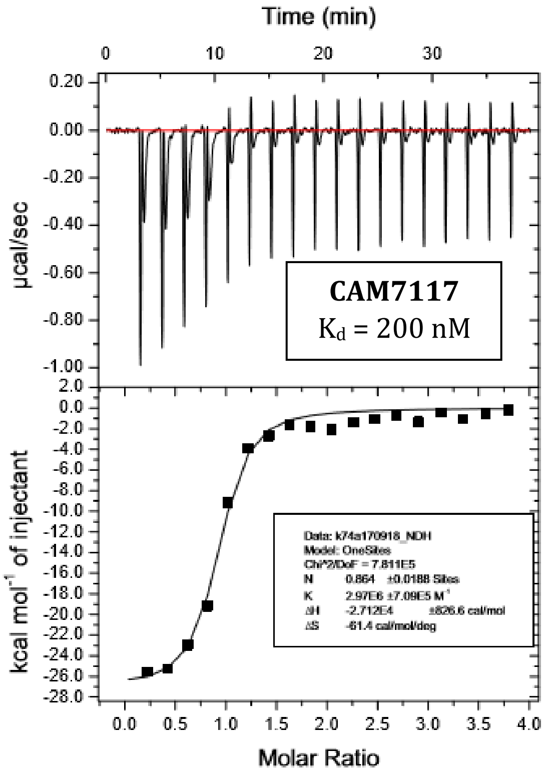
Time (min)
µcal/sec
CAM7117
Kd = 200 nM
kcal mol-1 of injectant
Data: k74a170918_NDH
Model: OneSites
Chi^2/DoF = 7.811E5
N 0.864 ±0.0188 Sites
K 2.97E6 ±7.09E5 M-1
ΔH -2.712E4 ±826.6 cal/mol
ΔS -61.4 cal/mol/deg
Molar Ratio

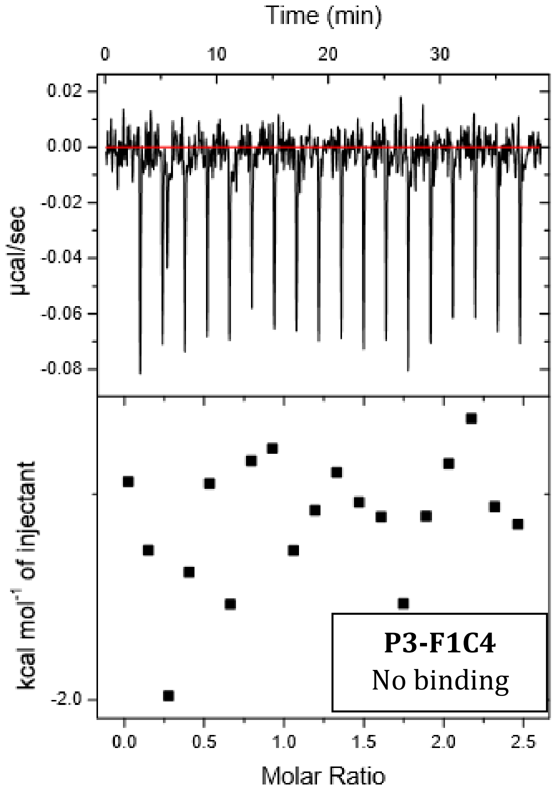
Time (min)
µcal/sec
kcal mol-1 of injectant
P3-F1C4
No binding
Molar Ratio

Figure S2

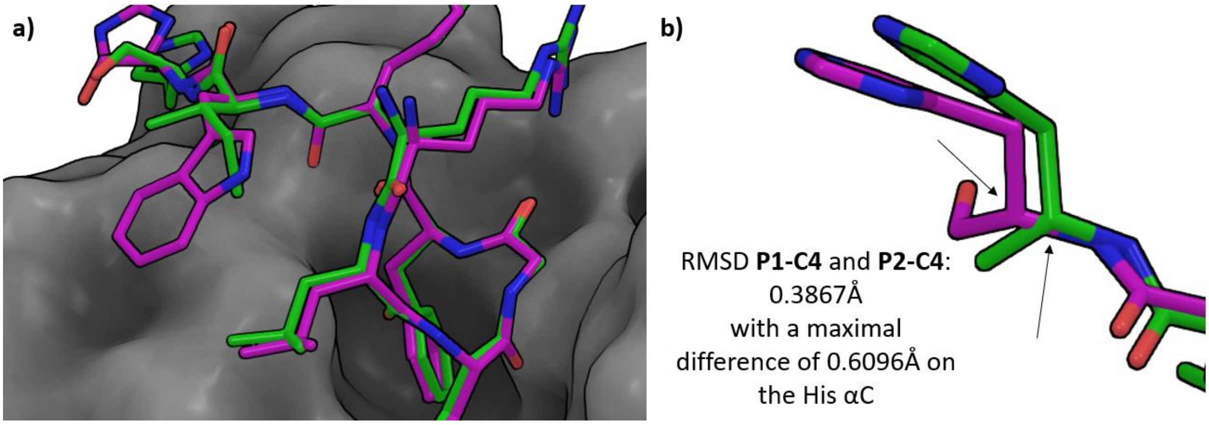

Figure S2 *– Comparison of the binding modes of P1-C4 and P2-C4. a) Overlay of the X-ray structure of P1-C4 (6Q38, green) and P2-C4 (purple, 6Q4Q) showing the two peptides adopt the same conformation upon binding CK2α. The I9W variation does not results in the Trp binding deeper in the pocket. b) RMSD of the two peptides was calculated and showed maximal, yet small, difference around the His αC atoms.*

Table S4

Table S4 *- Binding curves of CK2β in the presence of **P1-C4** and **P2-C4**.*

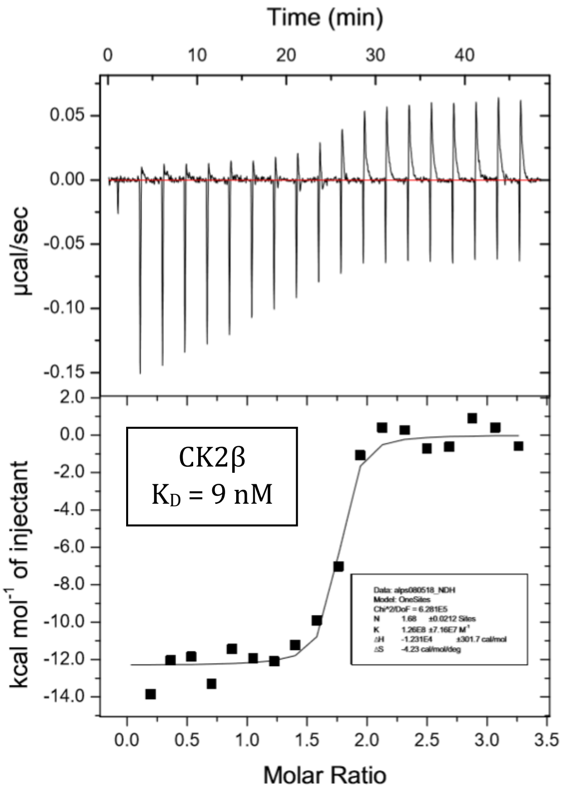

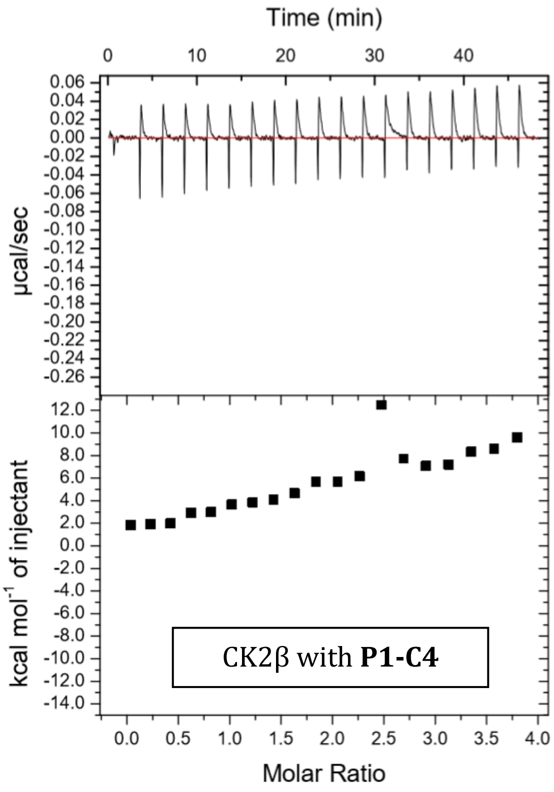

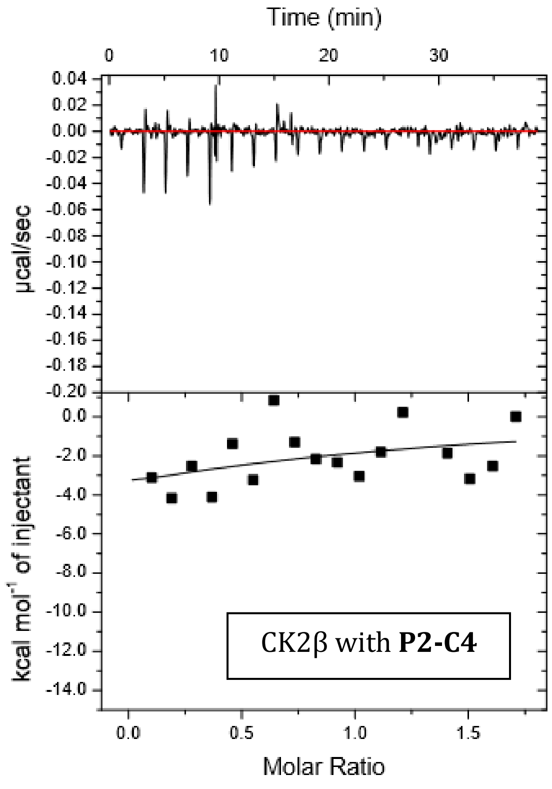

Figure S3

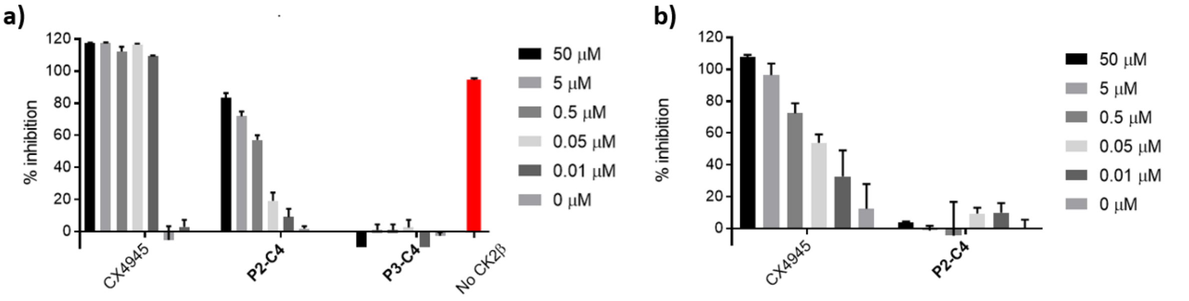

Figure S3 *– Inhibition of CK2β dependent and independent substrates. a) Inhibition of phosphorylation of eIF2b in the presence of CX4945 (positive control),* ***P2-C4*** *and the negative peptide* ***P3-C4****.In red, inhibition of the kinase activity towards eIF2b in the absence of CK2β The experiment was performed using the CK2α/β heterodimer. b) Inhibition of phosphorylation of a CK2β independent substrate (*RRRADDSDDDD*) in the presence of CX4945 (positive control) and* ***P2-C4.***

Scheme S1

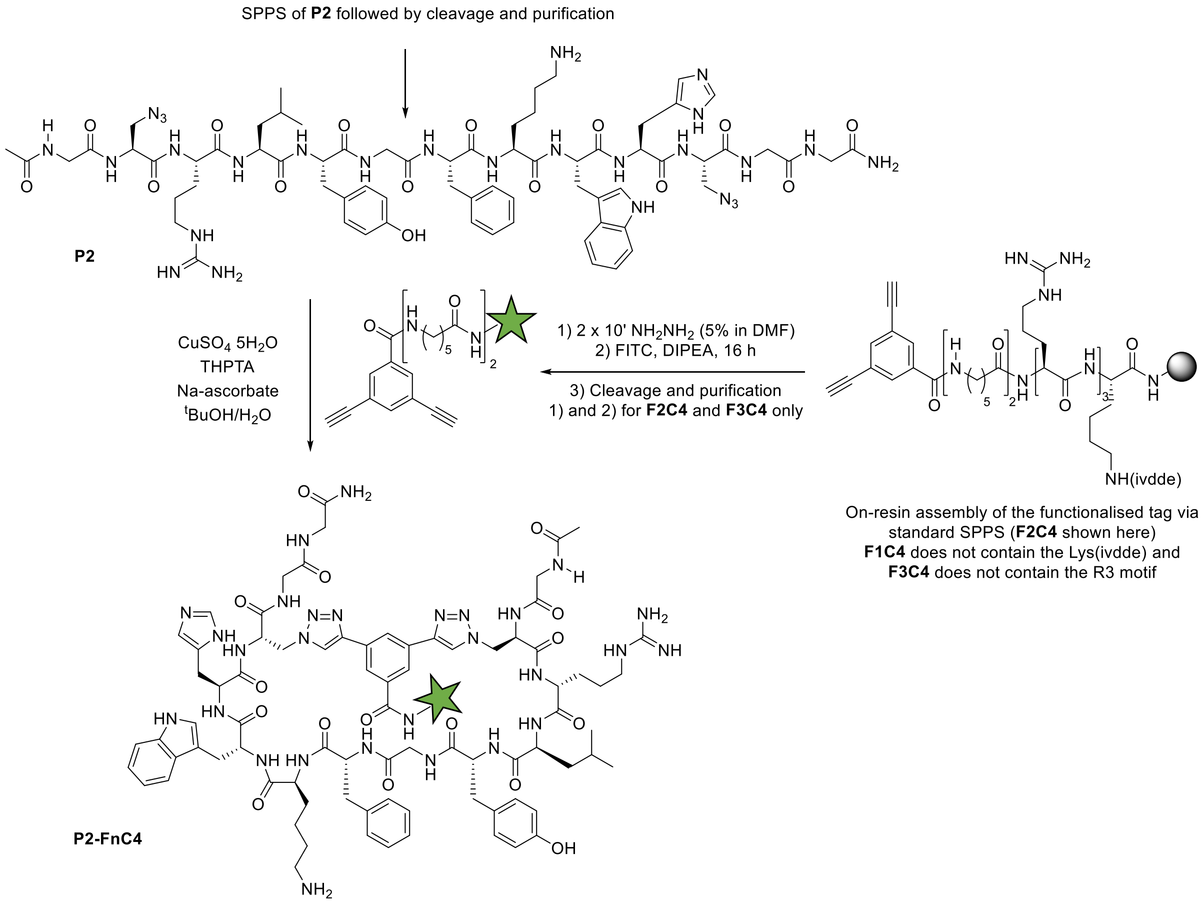

Scheme 1*- Fmoc-SPPS of peptide* ***P2****, functionalised constraints (****F1C4****,* ***F2C4****,* ***F3C4****) and stapling reaction. Green star indicates a generic functionality (****F1****,* ***F2*** *or* ***F3****).*

Figure S4

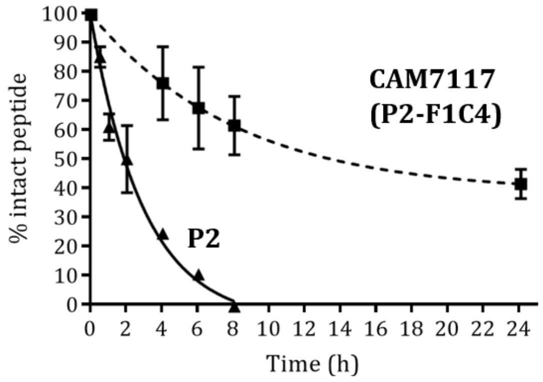

Figure S4 *- Serum stability test of the cyclic peptide* ***CAM7117*** *and the linear precursor* ***P2.***

Figure S5

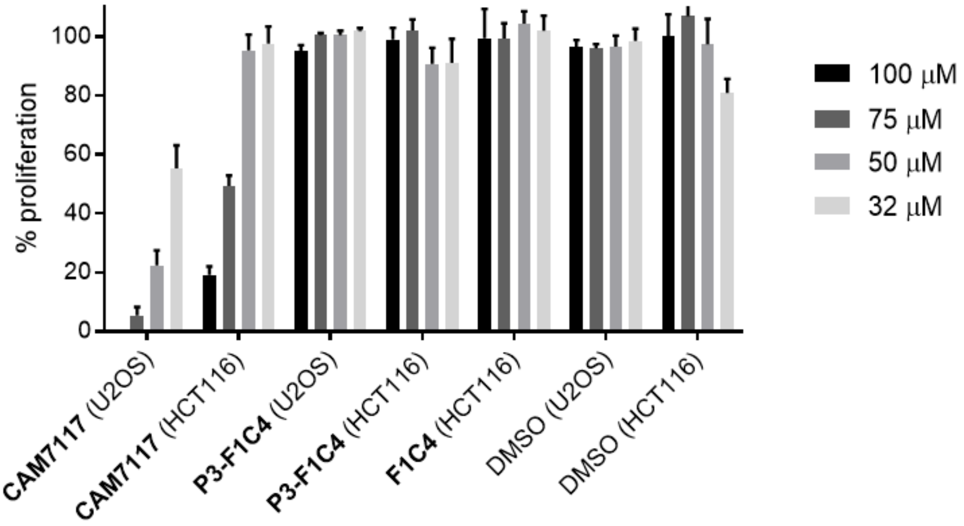

Figure S5 *- % proliferation of U2OS and HCT116 cells in the presence of high concentration of* ***CAM7117*** *and negative controls* ***P3-F1C4*** *(inactive, cell-permeable peptide),* ***F1C4*** *(cell-penetrating tag), and vehicle (DMSO).*

Figure S6

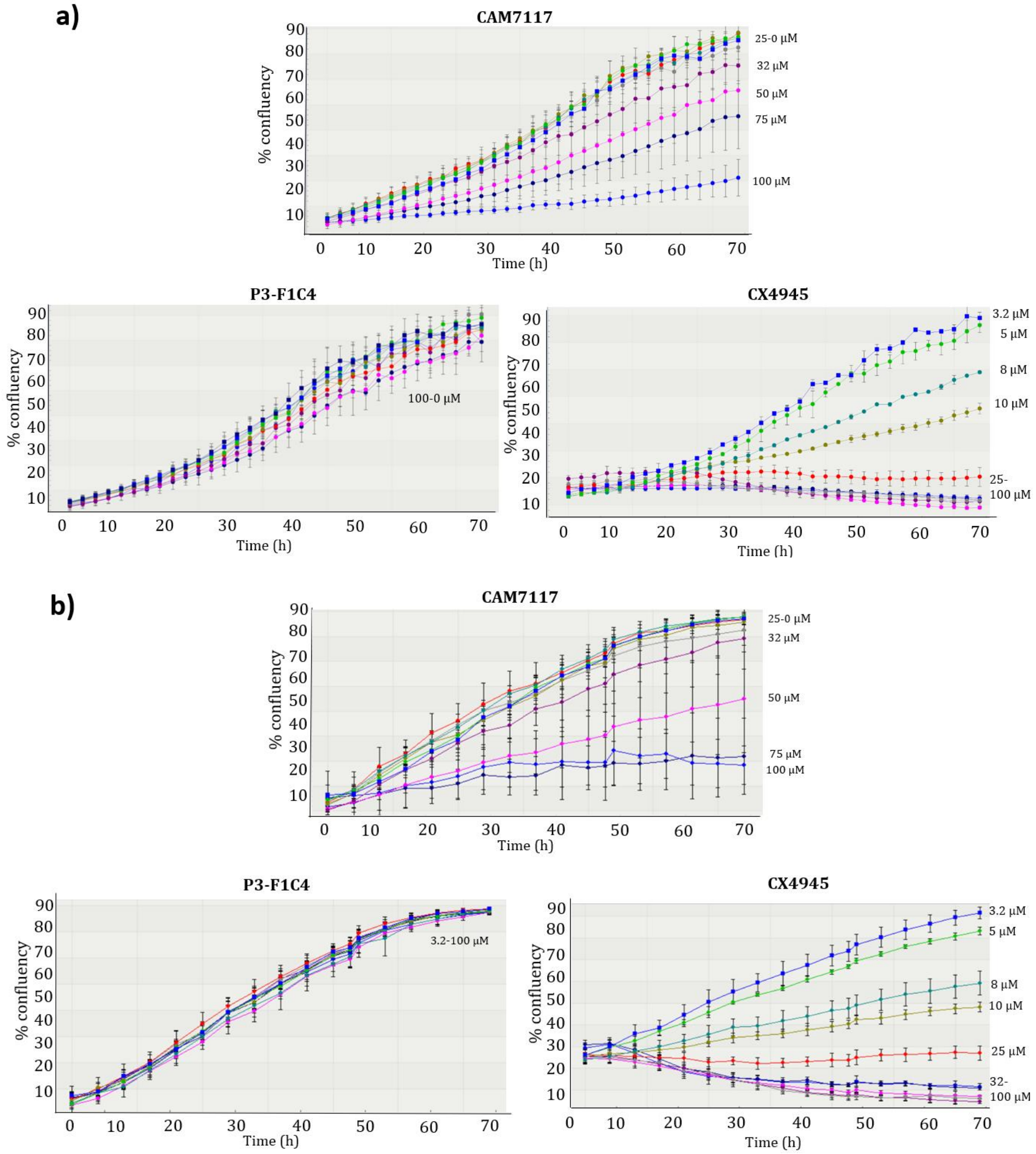

***Figure S6** – a) Rate of cell growth for **CAM7117**, negative peptide **P3-F1C4**, and positive control CX4945 in HCT116 cells; b) Rate of cell growth for **CAM7117**, negative peptide **P3-F1C4** and positive control CX4945 in U2OS cells.*

Figure S7

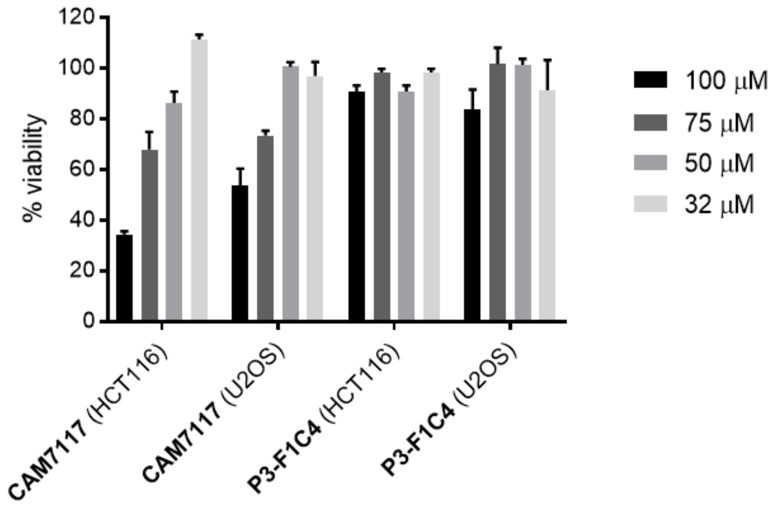

Figure S7 *- % viable cells after 4-hour treatment with the cell-permeable* ***CAM7117****, negative peptide* ***P3-F1C4*** *in HCT116 and U2OS cells.*

Figure S8

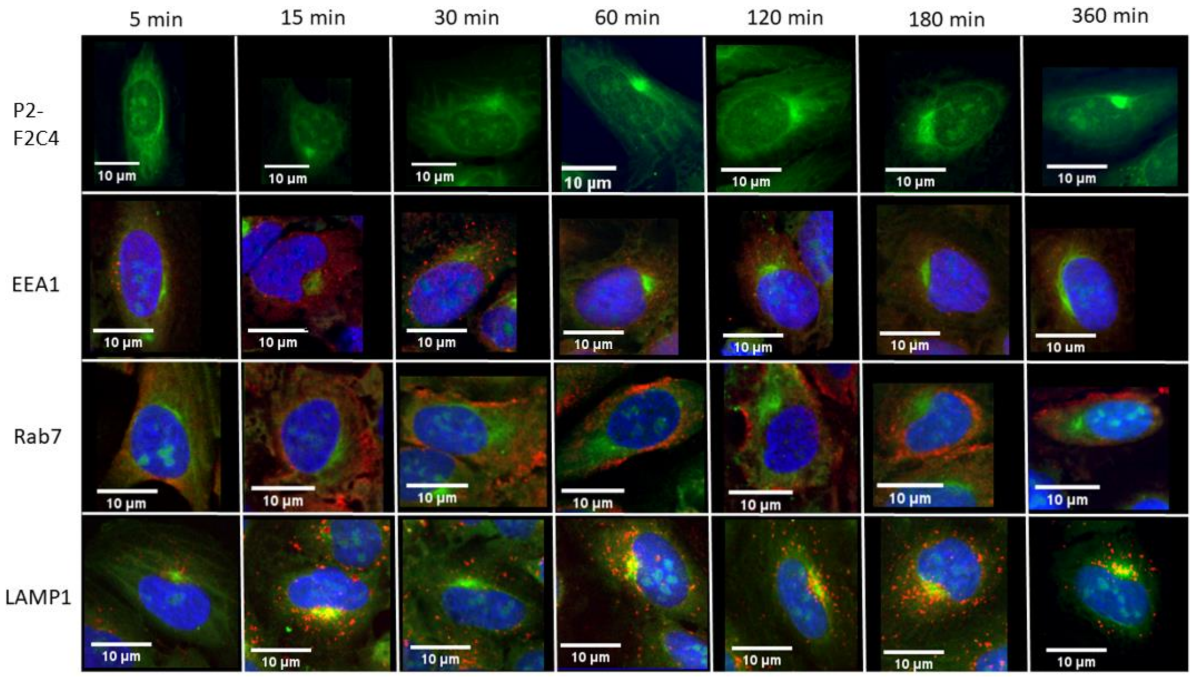

Figure S8 *–* ***P2-F2C4*** *co-localisation experiment with trafficking antibodies. Peptide is shown in green, antibodies in red and nuclei in blue.*

Figure S9

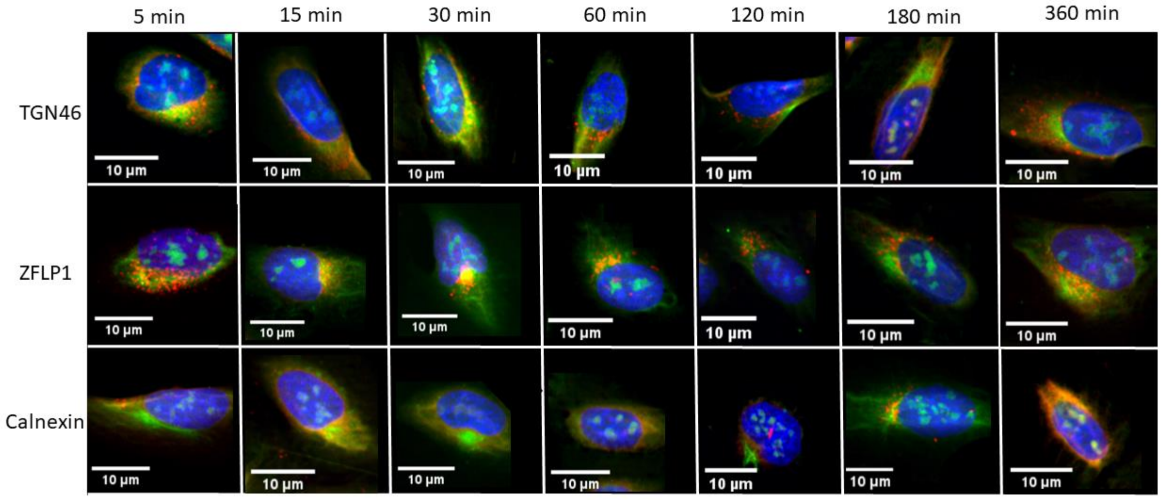

Figure S9* – *P2-F2C4 *co-localisation experiment with Golgi and ER stains. Peptide is shown in green, antibodies in red and nuclei in blue.*

1.2. Chemistry experimental

All experiments were carried out in oven-dried glassware under an atmosphere of N_2 using distilled solvents unless otherwise stated.

Reagents: Chemicals were purchased from commercial sources and used without further purification.

Yield: refer to chromatographically and spectroscopically pure compounds unless otherwise stated and are reported as follows: mass, moles, percentage

Temperature: Reaction temperatures of 0 °C were maintained using an ice-water bath and those of -78 °C using dry-ice and acetone; room temperature (rt) refers to 20-25 °C.

Flash chromatography: Analytical thin layer chromatography was carried out on SiO_2 Merck Kieselgel 60 F254 plates with visualisation either by ultraviolet light or staining with potassium permanganate or ninhydrin dips made using standard procedures. Retention factors (R_f) are quoted to 0.01. Flash column chromatography was performed using silica gel 60 (230-400 mesh), or standardised aluminium oxide 90 (150 mesh), under a positive pressure of N_2. Eluent systems are expressed in % v/v. NH_3 used in flash chromatography is a 7 N solution in MeOH.

Nuclear Magnetic Resonance (NMR): ^{1}H, ^{13}C and ^{19}F NMR spectra were recorded using an internal deuterium lock at ambient probe temperatures on the following instruments: Bruker Avance III 400 MHz HD Smart Probe Spectrometer, Bruker Avance III 400 MHz HD Spectrometer, Bruker 400 MHz QNP Cryoprobe Spectrometer, Bruker 500 MHz DCH Cryoprobe Spectrometer, Bruker Avance III 500 MHz HD Smart Probe Spectrometer. The following deuterated solvents were used: chloroform ($CDCl_3$), dimethylsulfoxide (DMSO-d_6) and methanol (CD_3OD). ^{1}H-NMR chemical shifts (δ) are quoted in ppm to the nearest 0.01 ppm, relative to the residual non-deuterated solvent peak and coupling constants (J) are quoted to the nearest 0.1 Hertz (Hz). ^{13}C-NMR chemical shifts are quoted to the nearest 0.1 ppm, relative to the solvent peak and coupling constants are quoted to the nearest 0.1 Hz. ^{19}F-NMR chemical shifts are quoted to the nearest 0.1 ppm. Spectral data is reported as follows: chemical shift, integration, multiplicity (s, singlet; d, doublet; t, triplet; q, quartet; sept, septet; m, multiplet; br, broad; or as a combination of these e.g. br s, dd, dt), coupling constant(s) and assignment. The numbering system used in the assignments does not necessarily follow the IUPAC convention. Assignment of all spectra is supported by DEPT, COSY, HSQC and HMBC or done by analogy to fully assigned spectra of closely related compounds.

Infra-red spectroscopy (IR): Infra-red spectra were recorded neat on a Perkin Elmer Spectrum One FT-IR spectrometer fitted with an Attenuated Total Reflectance (ATR) sampling accessory. Selected absorption maxima (ν_{max}) are quoted in wavenumbers (cm^{-1}) with the following abbreviations: w, weak; m, medium; s, strong; vs, br, broad.

Liquid chromatography-mass spectrometry (LCMS): LCMS was carried out using a Waters ACQUITY H-Class UPLC with an ESCi Multi-Mode Ionisation Waters SQ Detector 2 spectrometer using MassLynx 4.1 software; EI refers to the electrospray ionisation technique; LC system: solvent A: 2 mM NH_4OAc in H_2O/MeCN (95:5); solvent B: MeCN; solvent C: 2% formic acid; column: ACQUITY UPLC® CSH C18 (2.1 mm × 50 mm, 1.7 μm, 130 Å) at 40 °C; gradient: 5 – 95% B with constant 5% C over 1 min at flow rate of 0.6 mL/min; Injection volume: 5 μL. Chromatographs were monitored by absorbance using diode array detection at a wavelength range of 190-600 nm, interval 1.2 nm.

High resolution mass spectrometry (HRMS): HRMS was carried out using a Waters LCT Premier Time of Flight (ToF) mass spectrometer or the ThermoFinnigan Orbitrap Classic mass spectrometer. Reported mass values are within the error limits of ± 5 ppm mass units. ESI refers to the electrospray ionisation technique.

Analytical HPLC: Chromatographs were obtained on an Agilent 1260 Infinity using a Supelcosil ABZ+PLUS column (150 mm x 4.6 mm, 3 μm) eluting with a linear gradient system (solvent A: 0.05% (v/v) TFA in water, solvent B: 0.05% (v/v) TFA in MeCN) over 15 min, unless otherwise stated, at a flow rate of 1 mL/min. HPLC was monitored by UV absorbance at 220 and 254 nm.

Preparative HPLC: Preparative HPLC was carried out on an Agilent 1260 Infinity using a Supelcosil ABZ+PLUS column (250 mm x 21.2 mm, 5 μm) eluting with a linear gradient system (solvent A: 0.1% (v/v) TFA in water, solvent B: 0.05% (v/v) TFA in MeCN) over 20 min at a flow rate of 20 mL/min. HPLC was monitored by UV absorbance at 220 and 254 nm.

Automated Solid Phase Peptide Synthesis (SPPS): automated SPPS was carried out on solid-phase using a Fmoc-protecting group strategy on a CEM Liberty Blue Automated Microwave Peptide Synthesiser.

Microwave irradiation: Microwave irradiation was performed in a Biotage® microwave reactor.

Melting points: Melting points were measured using a Büchi melting point B545 apparatus and are uncorrected.

1.2.1. Experimental synthetic details

1.2.1.1. Small molecules

General method 1: TMS deprotection

Aqueous 6 M KOH (10 equiv) was added to a stirred solution of the TMS-protected alkyne (1 equiv) in MeOH (0.45 M). The mixture was stirred at rt for 18 hours. MeOH was removed under a stream of N_2 and the aqueous phase was acidified to pH 4 with HCl 6 N and extracted with EtOAc (3 x). The combined organic phases were dried ($MgSO_4$) and the solvent evaporated under reduced pressure to yield the desired compound.

Based on a literature procedure.[1]

General method 2: Addition of TMS-alkyne to esters and anhydride

Trimethylsilylacetylene (1.5-3 equiv) in anhydrous THF (1.2 M) was cooled down to -78 °C. ^{n}BuLi 1.6 M in hexane (3 equiv) was added dropwise and the reaction mixture was stirred at -78 °C for 1 hour. After this time the reaction was warmed to 0 °C for 10 minutes and then cooled down to -78°C. The appropriate benzoate or aldehyde (1 equiv) in anhydrous THF (1.1 M) was added over 10 minutes and the mixture stirred at rt for 2 hours. After this time the reaction was quenched with NH_4Cl saturated aqueous solution, THF removed under a stream of N_2 and the product extracted with Et_2O (3 x). The combined organic phases were dried ($MgSO_4$) and the residue purified by flash chromatography to give the desired product.

Based on a literature procedure.[2]

1.2.1.1.1. Unnatural amino acids

Imidazole-1-sulfonyl azide hydrogen Sulfate (1)

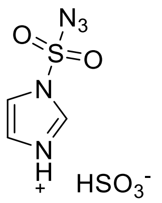

Sulfuryl chloride (16.1 mL, 200 mmol) was added dropwise to an ice-cold suspension of NaN_3 (13.0 g, 200 mmol) in MeCN (200 mL) and the mixture stirred for 16 hours. Imidazole (25.9 g, 380 mmol) was added and the pink mixture stirred at 0°C for 5 hours. The mixture was then diluted with EtOAc (400 mL) and H_2O (400 mL). The organic fraction was isolated and washed with H_2O (400 mL) and saturated $NaHCO_3$ (2 x 200 mL) and then dried ($MgSO_4$). The solvent was reduced to 200 mL under reduced pressure. A solution of conc. H_2SO_4 (11.0 mL) in EtOAc (100 mL) was added to the ice-cold reaction mixture over 30 min. The reaction mixture was warmed to room temperature and stirred for 16 hours. The precipitate was filtered off, washed with EtOAc (3 x 60 mL) and dried under reduced pressure to yield **1** as a white powder (34.95 g, 129 mmol, 64%).

$\boldsymbol{\delta_H}$ (400 MHz, DMSO-d_6): 14.27 (1H, s) 8.64 (1H, s), 7.98 (1H, app t, 1.6 Hz), 7.35 (1H, app q, *J* = 0.8 Hz), 7.09 (1H, s) (additional peaks can be seen at δ 9.09 and δ 7.69 due to decomposition in DMSO); $\boldsymbol{\delta_C}$ (101 MHz, DMSO-d_6): 138.2, 130.9, 119.6. **HRMS** (ESI+): *m/z* found $[M+H]^+$ 174.0073, $C_{18}H_{19}O_4N_2$ required 174.0080 (Δ 4.2 ppm).

Characterisation data in accordance with literature.[3]

Fmoc-Dap-OH (2)

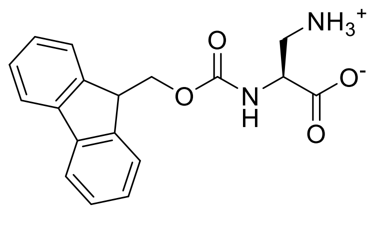

Fmoc-Asn-OH (4.00 g, 11.3 mmol) was added to a solution of [bis(trifluoroacetoxy)iodo]benzene (5.4 g, 16.95 mmol) in DMF/H_2O (2:1 53.2 mL: 26.4mL). After 15 minutes, pyridine (2.10 mL) was added and the mixture was stirred at rt for 16 hours. The solvent was removed under reduced pressure and the oily residue was dissolved in H_2O (60.0 mL). Concentrated HCl (2 mL) was added and the acidified solution was washed with Et_2O (3 x 40 mL). The aqueous phase was adjusted to pH 6 with 2 M NaOH solution and the resulting precipitate was filtered, washed with H_2O, ice-cold EtOH, Et_2O and

the residual solvent removed under reduced pressure to yield Fmoc-Dap-OH as a beige powder (2.99 g, 9.16 mmol, 81%).

δ_H (400 MHz, DMSO-d_6): 7.89 (2H, d, *J* = 7.4 Hz), 7.70 (2H, d, *J* = 6.2 Hz), 7.41 (2H, d, *J* = 7.4 Hz), 7.34 (2H, *J* = 7.4 Hz), 6.80 (1H, d, *J* = 6.2 Hz), 4.21-4.30 (3H, m), 3.71 (1H, m), 3.01 (1H, dd, *J* = 10.8, 4.8 Hz), 2.79 (1H, app t, *J* = 10.8 Hz); **δ_C** (101 MHz, DMSO-d_6): 171.5, 156.3, 144.3, 141.2, 128.1, 127.8, 125.8, 120.6, 66.2, 52.4, 47.1, 41.1; **HRMS** (ESI+): *m/z* found $[M+H]^+$ 327.1343, $C_{18}H_{19}O_4N_2$ required 327.1339 (Δ 1.2 ppm).

Characterisation data in accordance with literature.[4]

Fmoc-Aza-OH (3)

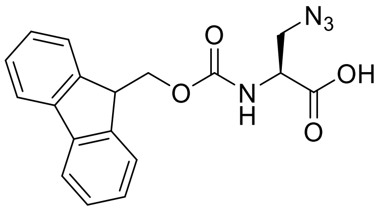

Fmoc-Dap-OH (4.04 g, 12.4 mmol) was added to a mixture of H_2O (60.0 mL), MeOH (180 mL), and CH_2Cl_2 (120 mL). $CuSO_4 \cdot 5\,H_2O$ (20 mg, 0.08 mmol) and imidazole-1-sulfonyl azide hydrochloride (8.08 g, 29.9 mmol) were added, the mixture adjusted to pH 9 with saturated K_2CO_3 and stirred for 18 hours. The mixture was then diluted with CH_2Cl_2 (120 mL), and the aqueous phase isolated. The organic phase was extracted with saturated $NaHCO_3$ (2 x 200 mL). The aqueous extract was washed with Et_2O (2 x 200 mL), acidified to pH 2 with conc. HCl, and extracted with Et_2O (3 x 240 mL). The organic extracts were dried ($MgSO_4$) and the solvent removed under reduced pressure. The oily residue was re-dissolved in EtOAc and the solvent removed under a stream of N_2 to yield **3** as a beige, amorphous solid (3.58 g, 9.53 mmol, 77%).

δ_H (400 MHz, DMSO-d_6): 7.95-7.87 (2H, m), 7.74 (2H, d, *J* = 7.4 Hz), 7.42 (2H, app t, *J* = 7.4 Hz), 7.32 (2H, app t, *J* = 7.4 Hz), 4.37-4.28 (2H, m), 4.24 (2H, m), 3.67-3.51 (2H, m); **δ_C** (101 MHz, DMSO-d_6): 171.5, 156.5, 144.2, 141.2, 128.1, 127.5, 125.7, 120.6, 66.3, 54.2, 51.3, 47.0; **$[\alpha]_D^{25}$** -9.9 (c = 1, DMF); **HRMS** (ESI+): *m/z* found $[M+H]^+$ 353.1248, $C_{18}H_{17}O_4N_4$ required 353.1244 (Δ 1.0 ppm).

Characterisation data in accordance with literature.[4]

1.2.1.1.2. Constraints

Methyl 3,5-bis((trimethylsylil)ethynyl)benzoate (4)

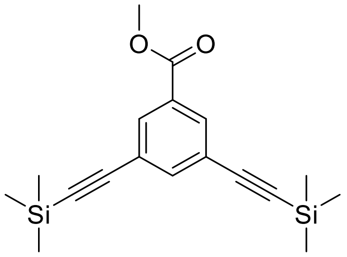

Trimethylsilyacetylene (6.00 mL, 42.2 mmol) was added to a stirring mixture of methyl 3,5-dibromobenzoate (900 mg, 3.10 mmol), $Pd_2(dba)_3$ (54.0 mg, 0.059 mmol), CuI (11.4 mg, 0.006 mmol) and PPh_3 (77.4 mg, 0.29 mmol) in dry triethylamine (15.0 mL). The reaction mixture was refluxed for 16 hours under N_2. The solvent was removed under reduced pressure, the residue was diluted with EtOAc and washed with H_2O. The organic phase was dried ($MgSO_4$) and the crude residue was purified via flash chromatography on silica gel (0-5% EtOAc in PE 40-60) to give **4** as a yellow oil (693 mg, 2.11 mmol, 68%).

R*f* =0.4 (5% EtOAc/PE 40-60); **Mp** = 73-75 °C; **δ_H** (400 MHz, $CDCl_3$): 8.06 (2H, app d, *J* = 1.44 Hz), 7.74 (1H, app t, *J* = 1.32 Hz), 3.92 (3H, s), 0.26 (18H, s); **δ_C** (101 MHz, $CDCl_3$): 165.7, 139.0, 132.6, 130.5, 123.9, 102.9, 96.1, 52.4, 0.2; **v_{max}**: 2956 (C≡C, m), 2157 (C≡C-H, m), 1729 (C=O, s); **HRMS** (ESI+): *m/z* found $[M+H]^+$ 329.1378, $C_{18}H_{25}O_3Si_2$ required 329.1388 (Δ 3.0 ppm).

Characterisation data in accordance with literature.[5]

3,5-diethylbenzoic acid (C4)

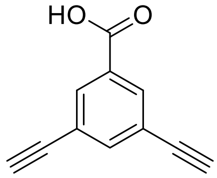

Prepared following *general method 2* using aqueous 6 M KOH (3.00 mL, 17.9 mmol), **4** (590 mg, 1.79 mmol), MeOH (4.00 mL). After the work-up, **C4** was obtained as an orange solid (300 mg, 1.76 mmol, 98%).

R*f* = 0.40 (20% MeOH/CH_2Cl_2); **δ_H** (400 MHz, CD_3OD): 8.05 (H, app d, *J* = 1.32 Hz), 7.72 (1H, app s), 3.66 (3H, s); **δ_C** (101 MHz, CD_3OD): 166.4, 138,5, 132.6, 131.6, 123.3, 81.0, 79.2; **v_{max}**: 3282 (C≡C, s), 1682 (C=O, s); **HRMS** (ESI+): *m/z* found $[M+H]^+$ 171.0444, $C_{11}H_7O_2$ required 171.0441 (Δ 1.9 ppm).

Characterisation data in accordance with literature.[1]

4-(3-hydroxy-1,5-bis(trimethylsilyl)penta-1,4-diyn-3-yl)benzoic acid (5)

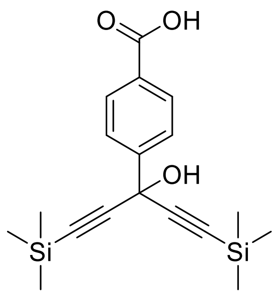

Prepared following *general method 3* using trimethylsilylacetylene (5.00 mL, 34.7 mmol) in THF (50.0 mL), ^{n}BuLi 1.6 M in hexane (25 mL, 36.0 mmol) and methyl-4-carboxybenzoate (2.50 g, 13.8 mmol) in THF (10.0 mL). After the work-up, the crude residue was purified by flash chromatography on silica gel (100% CH_2Cl_2) to yield **5** as an off white solid (2.20 g, 6.38 mmol, 46%).

R_f = 0.02 (0.5% MeOH/CH_2Cl_2); **Mp** = 199.8-200.6 °C **δ_H** (400 MHz, CD_3OD): 8.04 (2H, d, *J* = 8.4 Hz), 7.84 (2H, d, *J* = 8.4 Hz), 0.22 (18H, s); **δ_C** (101 MHz, CD_3OD): 168.0, 147.4, 130.4, 129.3, 125.6, 105.1, 88.6, 64.3, 1.7; **ν_{max}**: 3340 (O-H, br, w), 1683 (C=O, m); **HRMS** (ESI+): *m/z* found $[M+H]^+$ 345.1336, $C_{18}H_{25}O_3Si_2$ required 345.1342 (Δ -1.7 ppm).

4-(3-hydroxypenta-1,4-diyn-3-yl)benzoic acid (C5)

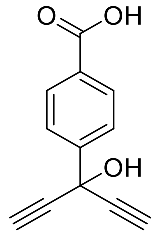

Prepared following *general method 2* using aqueous 6 M KOH (5.00 mL, 29.0 mmol), **5** (1.00 g, 2.84 mmol) and MeOH (6.00 mL). After the work-up, **C5** was yielded as a yellow solid (350 mg, 1.75 mmol, 62%).

R_f = 0.4 (20% CH_2Cl_2/MeOH); **Mp** = 154.4-155.6 °C; **δ_H** (400 MHz, CD_3OD): 8.06 (2H, d, *J* = 8.4 Hz), 7.88 (2H, d, *J* = 8.4 Hz), 3.23 (2H, s); **δ_C** (101 MHz, CD_3OD): 168.0, 147.2, 130.5, 129.3, 125.6, 83.6, 73.2, 63.5; **ν_{max}**: 3275 (C≡C, m), 1658 (C=O, s); **HRMS** (ESI+): *m/z* found $[M+Na]^+$ 223.0369, $C_{12}H_8O_3Na$ required 223.0366 (Δ 1.7 ppm).

1.2.1.1.3. ^{1}H and ^{13}C NMR spectra

1

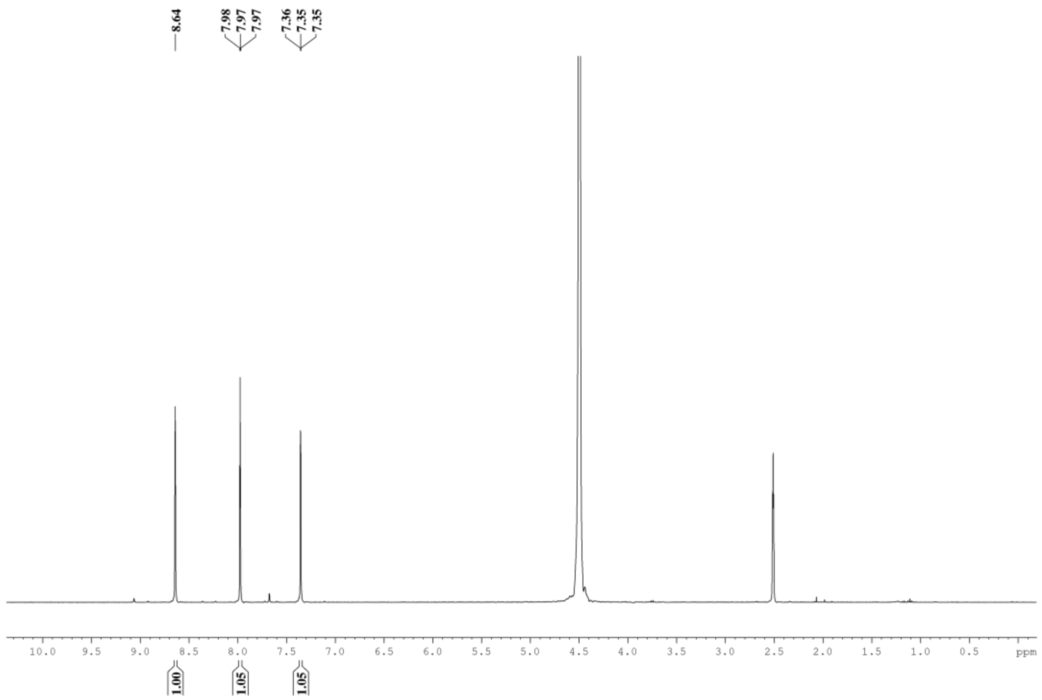

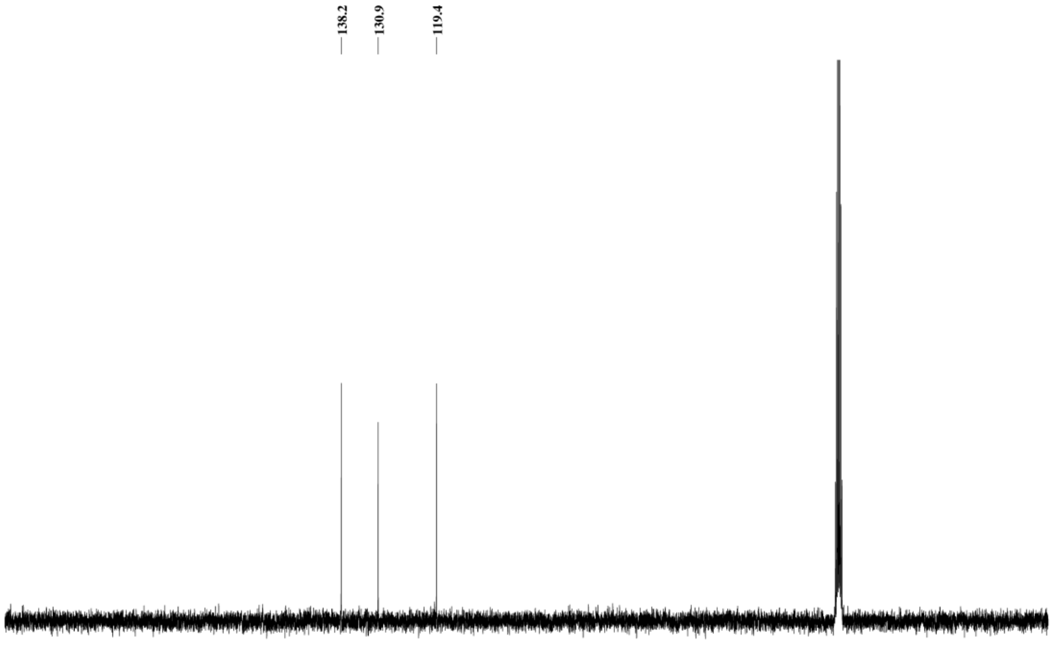

2

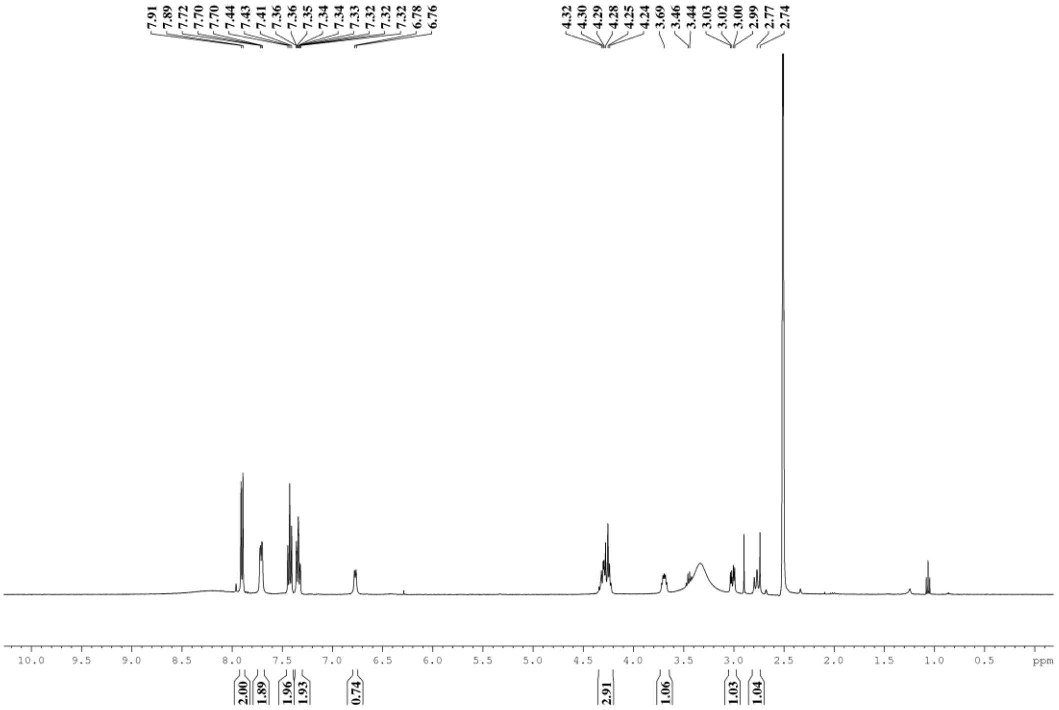
7.91
7.89
7.72
7.70
7.70
7.44
7.43
7.41
7.36
7.36
7.35
7.34
7.34
7.33
7.32
7.32
7.32
6.78
6.76
4.32
4.30
4.29
4.28
4.25
4.24
3.69
3.46
3.44
3.03
3.02
3.00
2.99
2.77
2.74
2.00
1.89
1.96
1.93
0.74
2.91
1.06
1.03
1.04
ppm

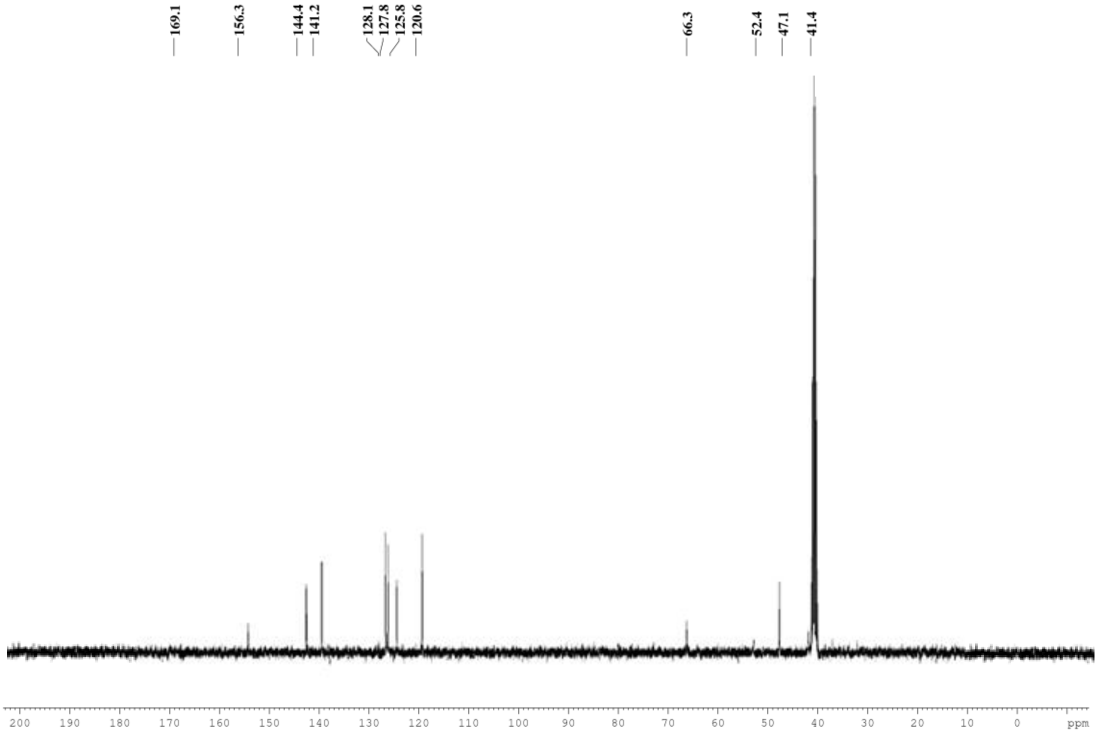
169.1
156.3
144.4
141.2
128.1
127.8
125.8
120.6
66.3
52.4
47.1
41.4
ppm

3

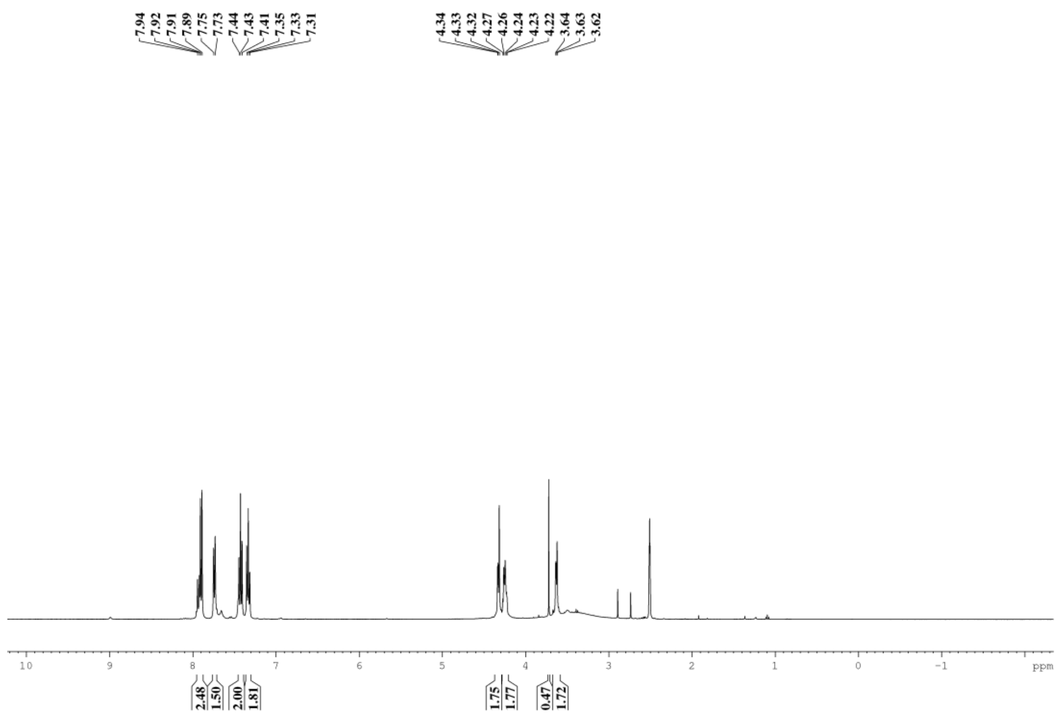
7.94
7.92
7.91
7.89
7.75
7.73
7.44
7.43
7.41
7.35
7.33
7.31
4.34
4.33
4.32
4.27
4.26
4.24
4.23
4.22
3.64
3.63
3.62
2.48
1.50
2.00
1.81
1.75
1.77
0.47
1.72
ppm

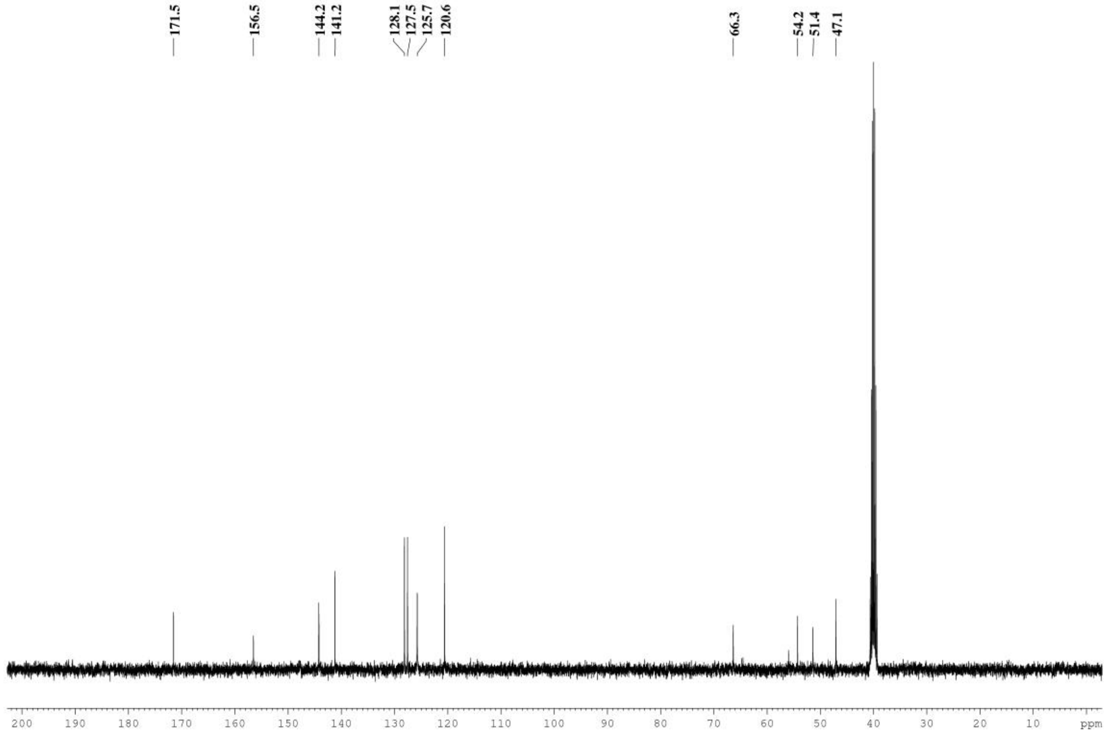
171.5
156.5
144.2
141.2
128.1
127.5
125.7
120.6
66.3
54.2
51.4
47.1
ppm

4

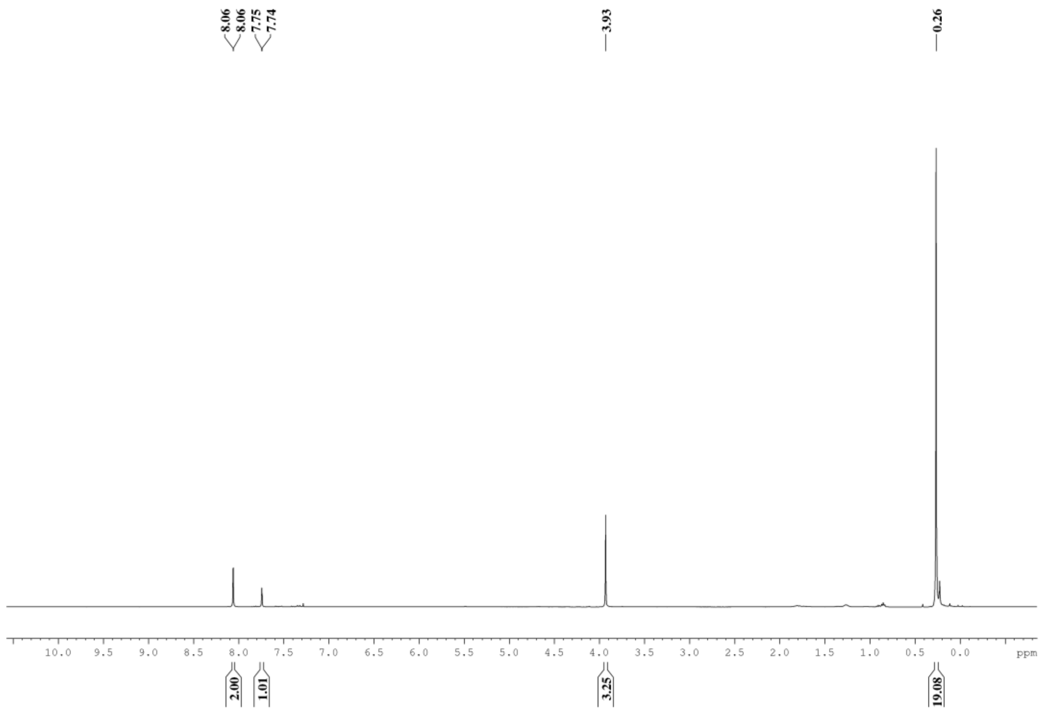
8.06
8.06
7.75
7.74
3.93
0.26
10.0 9.5 9.0 8.5 8.0 7.5 7.0 6.5 6.0 5.5 5.0 4.5 4.0 3.5 3.0 2.5 2.0 1.5 1.0 0.5 0.0 ppm
2.00
1.01
3.25
19.08

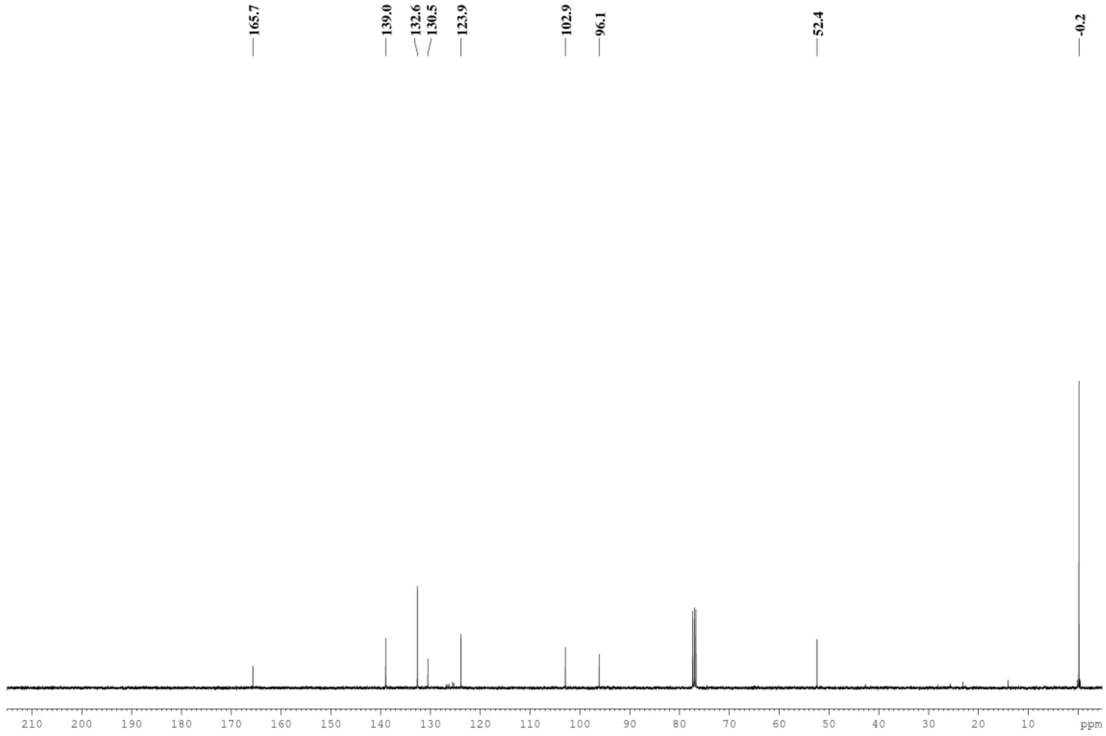
165.7
139.0
132.6
130.5
123.9
102.9
96.1
52.4
-0.2
210 200 190 180 170 160 150 140 130 120 110 100 90 80 70 60 50 40 30 20 10 ppm

C4

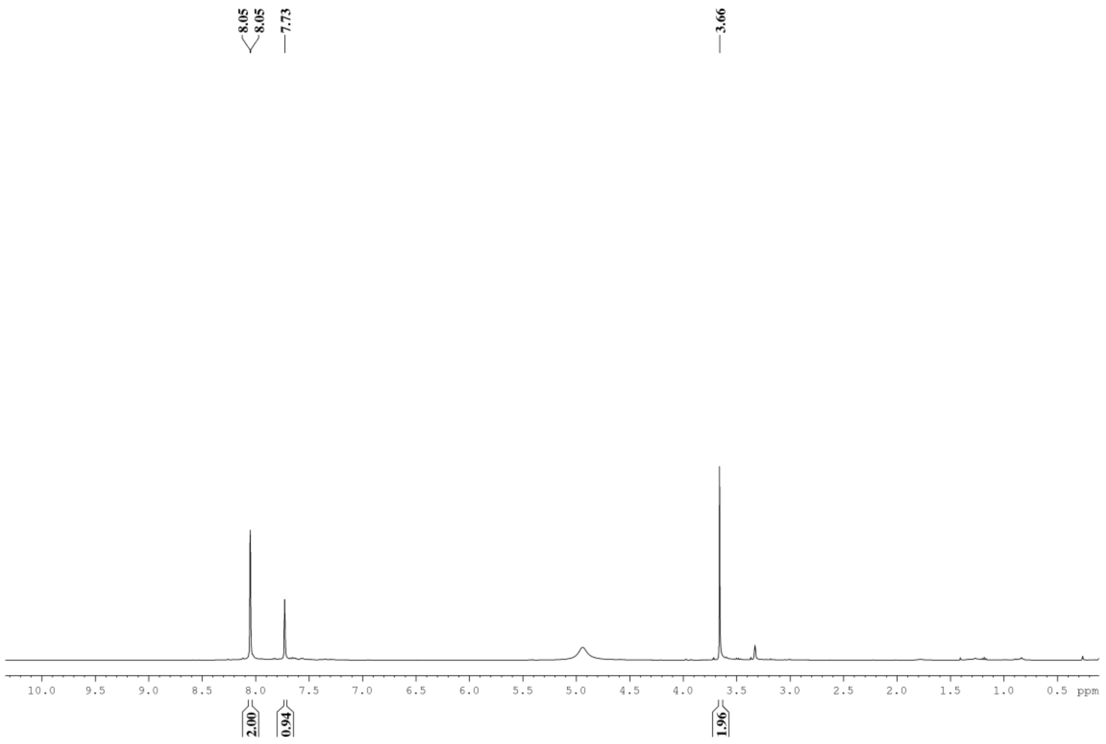

8.05
8.05
7.73
3.66
10.0 9.5 9.0 8.5 8.0 7.5 7.0 6.5 6.0 5.5 5.0 4.5 4.0 3.5 3.0 2.5 2.0 1.5 1.0 0.5 ppm
2.00
0.94
1.96

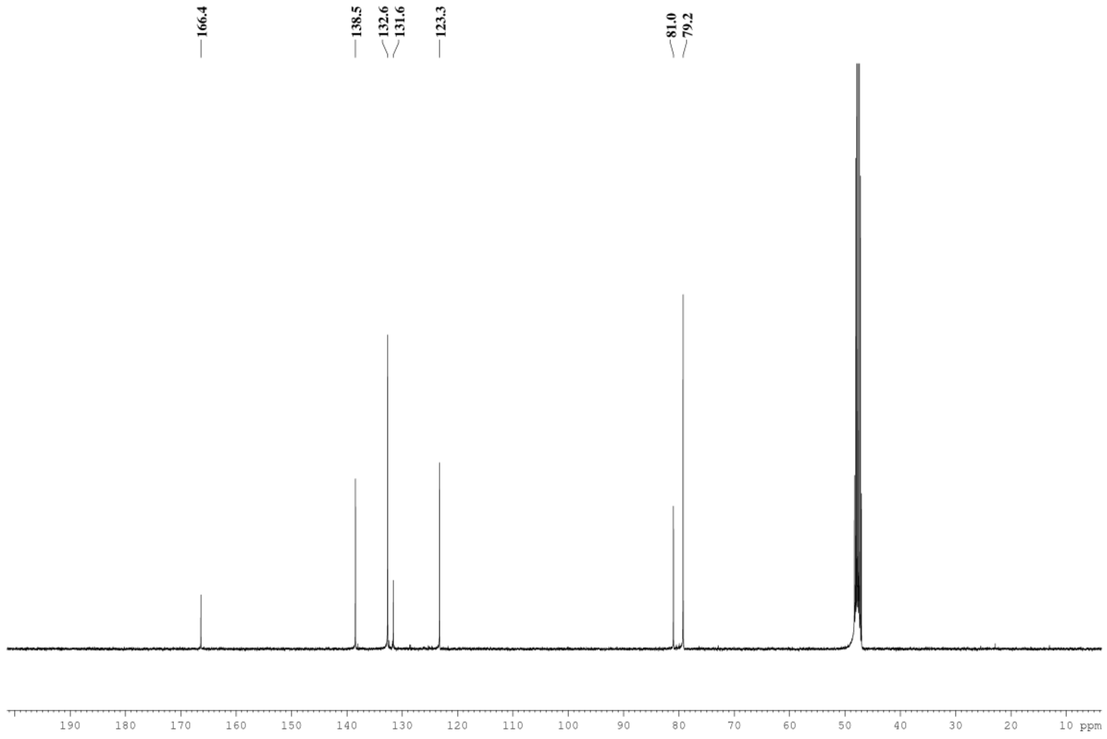

166.4
138.5
132.6
131.6
123.3
81.0
79.2
190 180 170 160 150 140 130 120 110 100 90 80 70 60 50 40 30 20 10 ppm

5

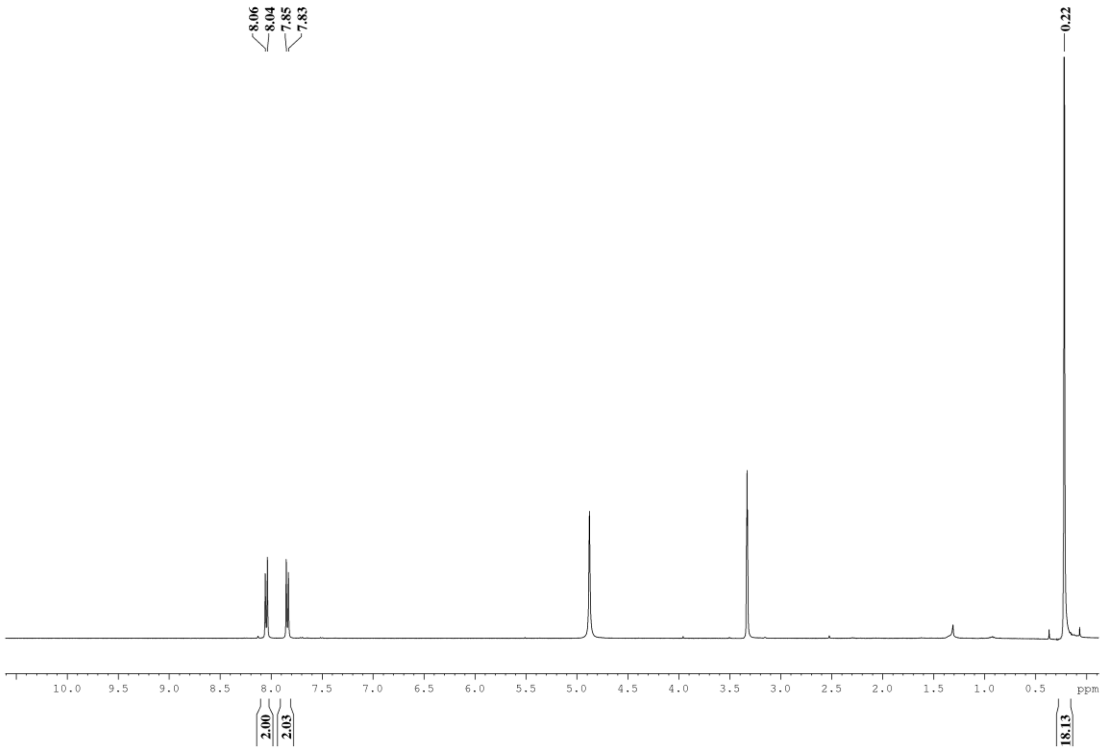
8.06
8.04
7.85
7.83
0.22
2.00
2.03
18.13
ppm

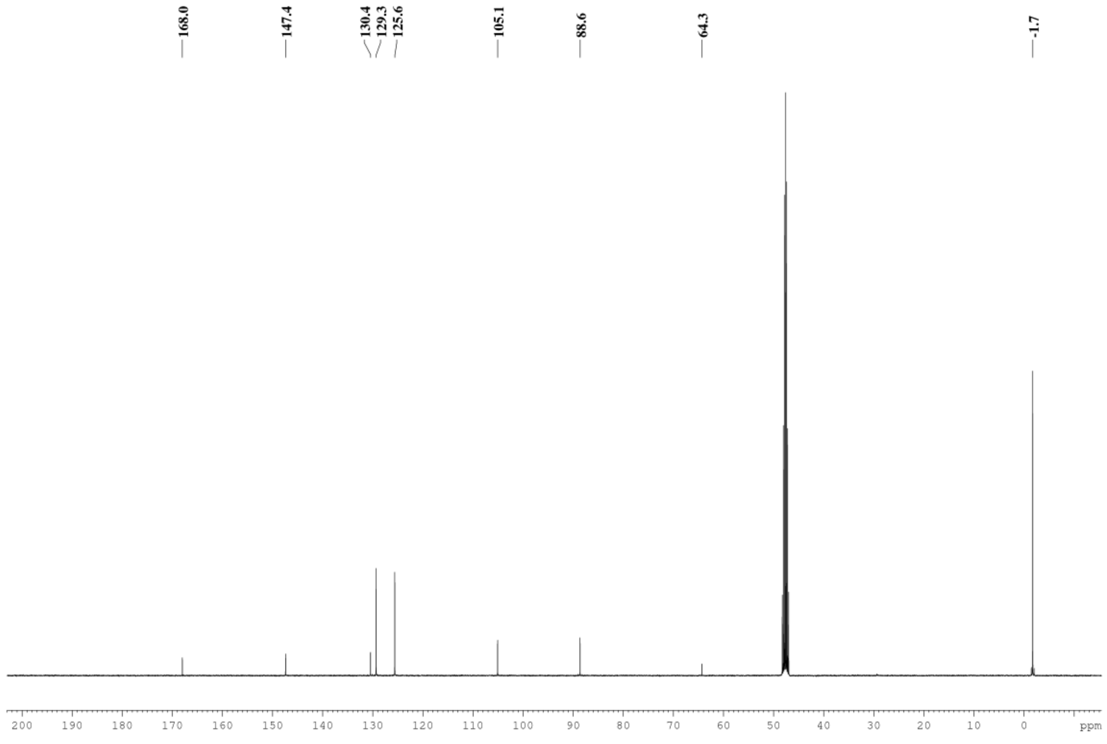
168.0
147.4
130.4
129.3
125.6
105.1
88.6
64.3
-1.7
ppm

C5

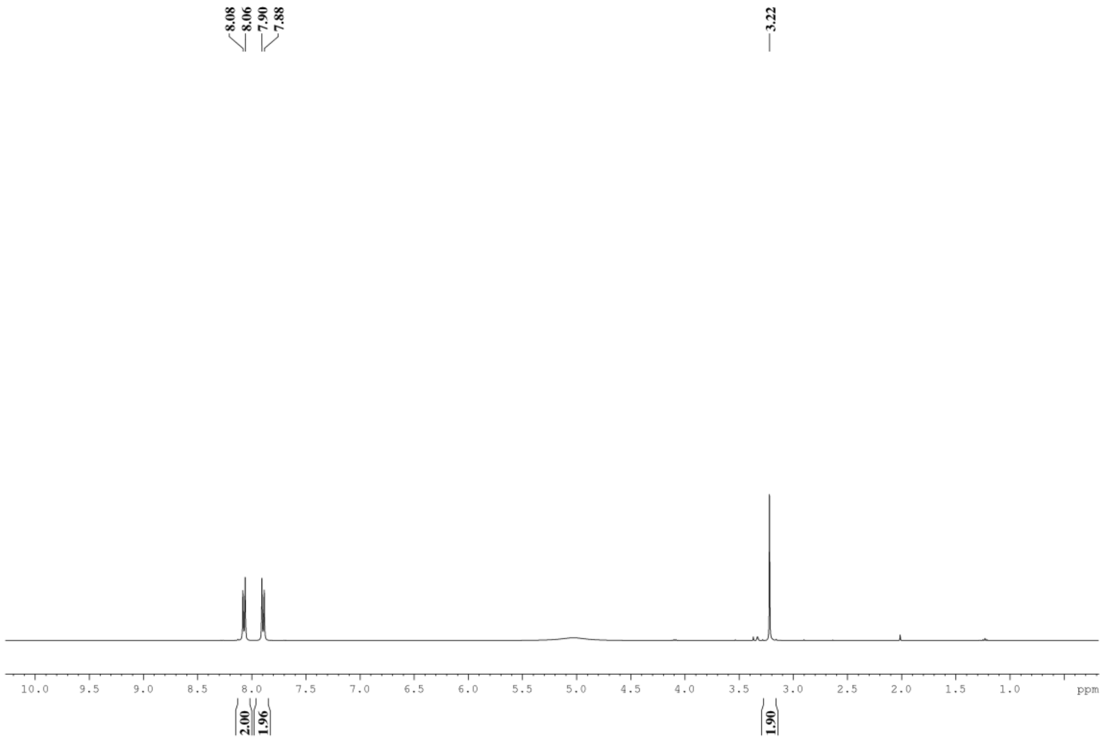
8.08
8.06
7.90
7.88
3.22
2.00
1.96
1.90
ppm

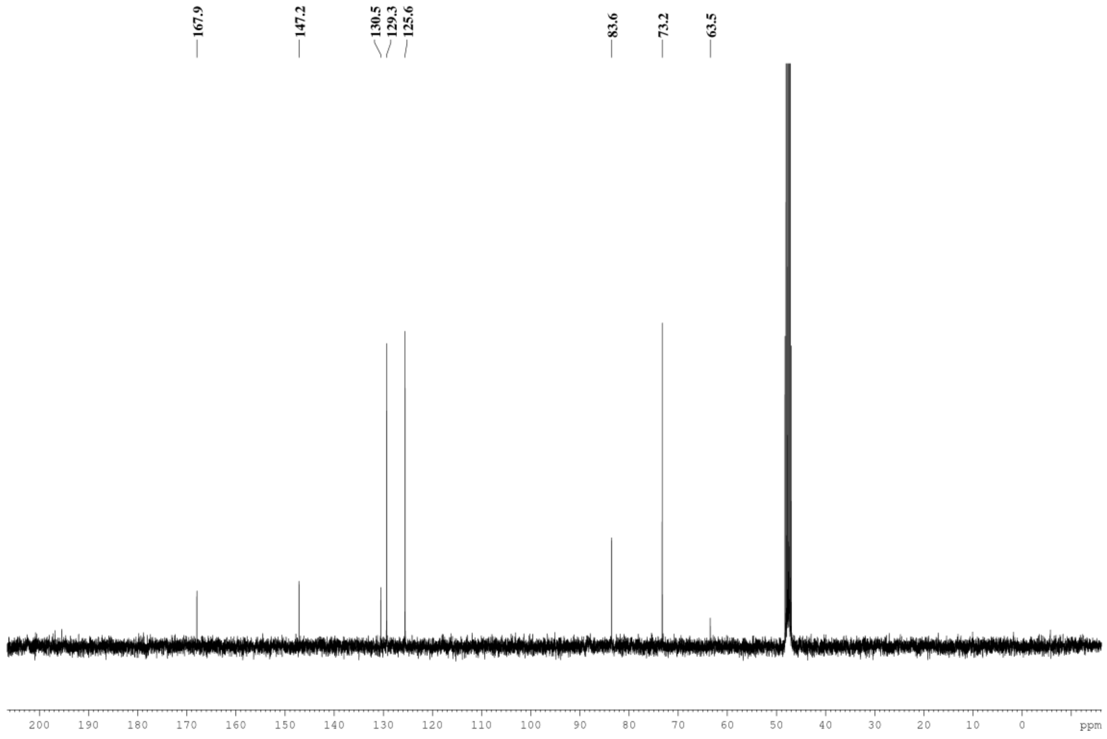
167.9
147.2
130.5
129.3
125.6
83.6
73.2
63.5
ppm

1.2.1.2. Peptides

Manual peptide synthesis

Manual peptide synthesis was performed on Merck LL Rink amide resin (0.33 mmol/g). Couplings were carried out by adding HATU (4 equiv) to a solution of the Fmoc-protected amino acid (4 equiv) in DMF (~0.4 M). After 10 seconds, the mixture was added to DIPEA (8 equiv). This pre-activated mixture was then added to the resin in DMF and shaken for 3 minutes. The coupling time was extended in the case of N-terminal capping with **C4** (1 hour) and coupling of Arg and unnatural amino acids (30 minutes). The side chain protecting groups used were: tBu for Asp, Glu, Ser, Thr, Tyr; Boc for Lys, Trp; Pbf for Arg; Trt for Asn, Gln, His. Fmoc-Lys(ddve)-OH was used for conjugation of FITC with Lys. Fmoc-Gly(Dmb)-OH was used instead of Fmoc-Gly-OH for the synthesis of peptide **P2**.

Fmoc deprotection was carried out with 20% piperidine in DMF (3 x 3 minutes).

N-terminal capping with FITC and Ac_2O (2 equiv) was achieved using DIPEA (4 equiv) in CH_2Cl_2 overnight (FITC) or for 1 hour (Ac_2O).

On-resin attachment of FITC via Lys was achieved by orthogonal deprotection of the Lys(ddve) with 5% NH_2NH_2 in DMF (2 x 10 minutes) followed by conjugation with FITC (2 equiv) in the presence of DIPEA (4 equiv) overnight.

Completion of amide couplings and Fmoc deprotection was determined by a chloranil test, in which acetaldehyde (200 µL) and a saturated solution of chloranil in toluene (50 µL) were added to a small amount of resin swelled in CH_2Cl_2. After 10 seconds shaking at rt, no change in colour indicated complete coupling, whilst green colouration of the resin indicated presence of a free amine. Any incomplete couplings was submitted to a second round of coupling.

Side chain deprotection and cleavage from the resin was achieved with TFA containing 2.5% TIPS and 2.5% H_2O for 3 hours at rt or 1 hour at 42 °C. In case of methionine and cysteine-containing peptides, cleavage was achieved with TFA containing 5% EDT, 5% H_2O and 2.5% TIPS. After cleavage, the mixture was filtered through a sintered funnel, the beads washed with MeOH and the filtrate was concentrated under a stream of N_2. The crude residue was triturated with cold Et_2O before purification by preparative HPLC.

Automated Fmoc solid-phase peptide synthesis

Automated peptide synthesis was carried out on a CEM Liberty Automated Microwave Peptide Synthesiser using Merck LL Rink Amide resin (0.33 mmol/g). All peptide couplings were performed with Fmoc-protected amino acids (5 equiv), Oxyma pure (10 equiv) and DIC (5 equiv) in DMF. Arg was coupled using double couplings for 15 min each without microwave irradiation. All other amino acids were coupled with 25 W power at 75 °C over 15 min.

Fmoc deprotection was achieved with a solution of 20% piperidine in DMF, using 45 W power at 75 °C over 3 min. N-terminal capping, cleavage and HPLC purification of peptides were carried out as previously described for manual SPPS.

Peptide macrocyclisation via Copper-catalysed azido-alkyne click (CuAAC)

A solution of diazido peptide (1 equiv) and dialkynyl linker (1 equiv) in 1:1 $^{t}BuOH/H_2O$ (0.8 mL/mg peptide) was degassed with N_2 for 15 min, followed by the addition of $CuSO_4 \cdot 5\ H_2O$ (1 equiv), THPTA (1 equiv) and sodium ascorbate (3 equiv). All the reactions were stirred under N_2 and monitored by LCMS. When the conversion reached > 80% the reaction mixture was diluted with H_2O and lyophilised prior purification. The absence of azido peak on IR (~ 2100 cm^{-1}) was checked for all the purified, dried peptide.

Based on a literature procedure.[6]

Peptide macrocyclisation via disulphide bridge formation

To a solution of the linear peptide in aqueous AcOH (50%) was added 2 mM I_2 in MeOH dropwise. After 5 minutes the reaction was quenched by adding ascorbic acid (1 M). The crude mixture was directly purified on preparative HPLC.

Based on a literature procedure.[7]

1.2.1.2 Peptide characterisation

1.2.1.2.1.1. Conversions of CuAAC peptide macrocyclisation

The table below lists the conversions observed via LCMS for the CuAAC peptide macrocyclisations reported in this work.

Table S5 *- LCMS conversion of peptides that underwent CuAAC cyclisation*

Peptide	Conversion (%)
**P0-C1**	82
**P1-C2**	79
**P1-C3**	62
**P1-C4**	52
**P1-C5**	92
**P1-F1C4**	79
**P1-F2C4**	54
**P2-C4**	83
**P1-F3C4**	87
**CAM7117**	60
**P2-F2C4**	72

1.2.1.2.1.2. LCMS and purity of CK2 peptides

Table S6*- Sequence, mass, purity and retention time of CK2 peptides*

Peptide	Sequence	Mass	*m/z* found	*m/z* calcul.	Species	Purity	Rt*
**Pc**	Ac-GCRLYGFKIHGCG-NH_2 (S-S)	1448.7	1449.5	1449.7	M+H	87%	7.26
**Pc linear**	Ac-GCRLYGFKIHGCG-NH_2	1450.7	1451.49	1451.7	M+H	99%	9.62
**Pc short linear**	Ac-CRLYGFIHGC-NH_2	1336.6	1337.3	1337.7	M+H	90%	9.92
**Pc short**	Ac-CRLYGFKIHGC-NH_2 (cyclic)	1334.6	1335.4	1335.6	M+H	84%	14.32
**P0-C1**	Ac-XRLYGFKIHX-NH_2 + C1	1376.7	1377.5	1377.7	M+H	97%	6.96
**P1-C3**	Ac-GXRLYGFKIHXGG-NH_2 + C3	1532.8	1533.6	1533.8	M+H	97%	6.98
**P1-C2**	Ac-GXRLYGFKIHXGG-NH_2 + C2	1423.7	1424.5	1424.7	M+H	97%	7.85
**P0**	Ac-XRLYGFKIHX-NH_2	1297.7	1298.9	1298.7	M+H	> 99%	10.16
**P1-C4**	Ac-GXRLYGFKIHXGG-NH_2 + C4	1638.9	1639.4	1639.9	M+H	89%	7.46
**P1-C5**	Ac-GXRLYGFKIHXGG-NH_2 +C5	1671.0	836.2	836.2	M+2H	92%	7.14
**P1**	Ac-GXRLYGFKIHXGG-NH_2	1468.7	1470	1469.8	M+H	91%	7.29
**eIF2β**	Ac-MSGDEMIFDPTMS$(K)_8$P-NH_2	1311.7	1312.9	1312.7	M+2H	90%	6.85
**P1-F1C4**	Ac-GXRLYGFKIHXGG-NH_2+ F2C4	2334.3	1168.1	1168.1	M+2H	>99%	6.96
**P1-F2C4**	Ac-GXRLYGFKIHXGG-NH_2+ F1C4	2851.4	1427.3	1426.7	M+2H	98%	7.69
**P2**	Ac-GXRLYGFKWHXGG-NH_2	1543.7	1544	1544.7	M+H	95%	7.48
**P2-C4**	Ac-GXRLYGFKWHXGG-NH_2 + C4	1713.6	1714.7	1714.6	M+H	>99%	7.29
**P1-F3C4**	Ac-GXRLYGFKIHXGG-NH_2 + F3C4	1191.1	1192.7	1192.1	M+2H	89%	8.44
**CAM7117**	Ac-GXRLYGFKWHXGG-NH_2 +F2C4	2407.3	1205	1204.5	M +2H	>99%	7.22

**P2-F2C4**	Ac-GXRLYGFKWHXGG-NH_2 +F1C4	2929.5	1462.2	1463.7	M+2H	95%	7.62

* rt (retention time) on a 5 to 95 % B over 15 minutes on analytical HPLC

1.2.1.2.1.3. LCMS and purity of CPP constraints

Table S7*-Sequence, mass, purity and retention time of cell-penetrating peptides*

Peptide	Sequence	Mass	*m/z* found	*m/z* calcul.	Species	Purity	Rt*
**F1C4**	**C4**-$(Ahx)_2$+[(D)R]$_3$$NH_2$	863.5	864.9	864.5	M+H	99%	7.47
**F2C4**	**C4**-Ahx_2[(D)R]$_3$K-FITC-NH_2	1381.7	1382.4	1382.7	M+H	83%	9.18
**F3C4**	**C4**-$(Ahx)_2$+K+FITC-NH_2	911.3	912.4	911.3	M+H	97%	7.18

* rt (retention time) on a 5 to 95 % B over 15 minutes on analytical HPLC

1.2.1.2.1.4. Purification gradients

The table below lists the gradient used in the purification of the peptides on preparative HPLC. Gradient on the right column shows the initial and final percentage of MeCN (0.05% TFA) in H_2O (0.1% TFA) over 20 minutes.

Table S8 *- Purification gradients*

Peptide	Gradient
**P0**	5-95
**P1**	15-50
**P0-C1**	5-95
**P1-C2**	5-60
**P1-C3**	5-60
**P1-C4**	10-50
**P1-C5**	5-55
**P2**	10-60
**P1-F1C4**	5-60
**P1-F3C4**	5-95
**P2-C4**	15-70
**F1C4**	30-70
**F2C4**	3-60
**F3C4**	5-95
**P2-F2C4**	15-70
**CAM7117**	5-60

1.2.1.2.1.5. HPLC traces of selected peptides

Table S9 *- Analytical HPLC traces of selected peptides*

Pc

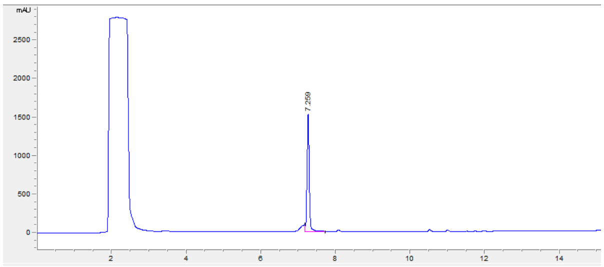

Short Pc

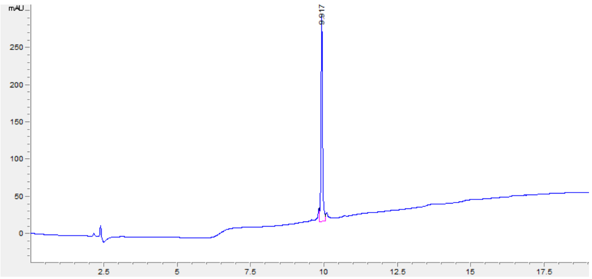

P0-C1

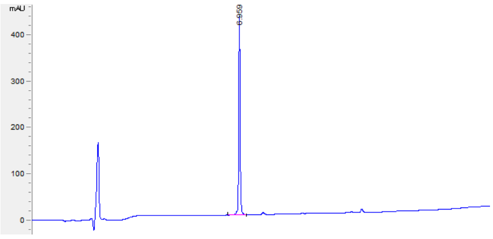

P1-C2

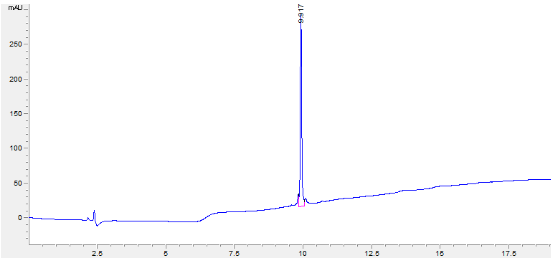

P1-C3

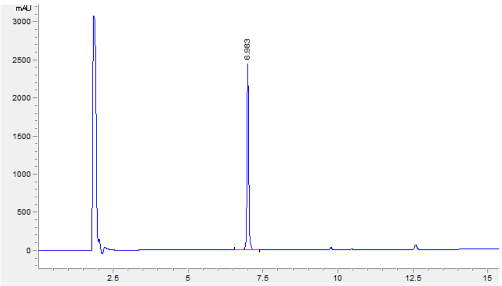

P1-C4

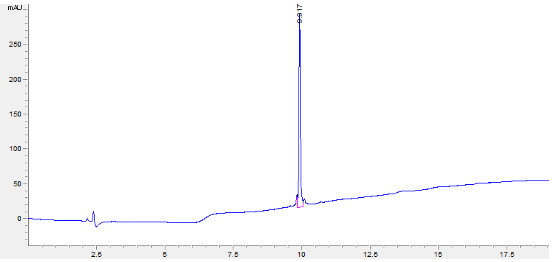

P1-C5

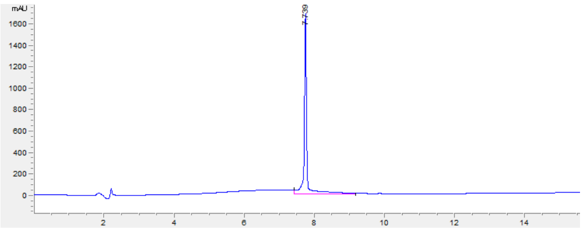

P3-F1C4

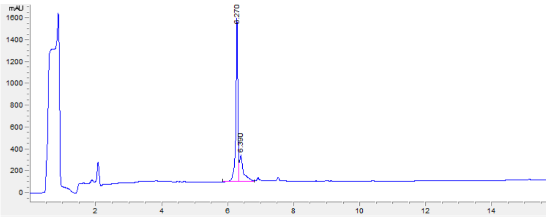

P1-F1C4

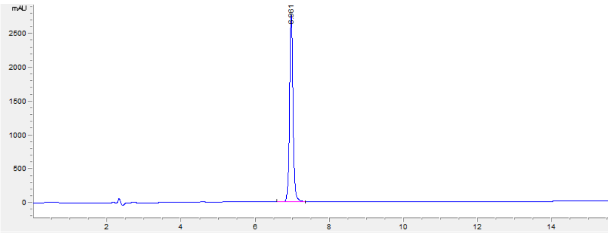

P1-F2C4

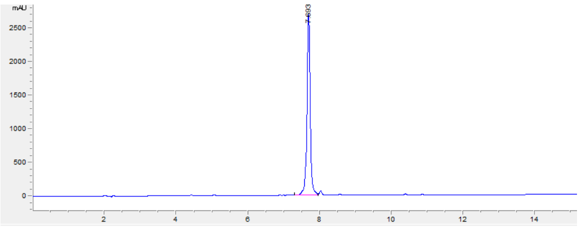

P2

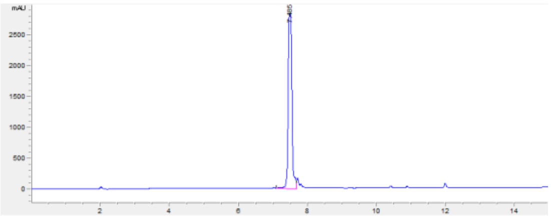

P2-C4

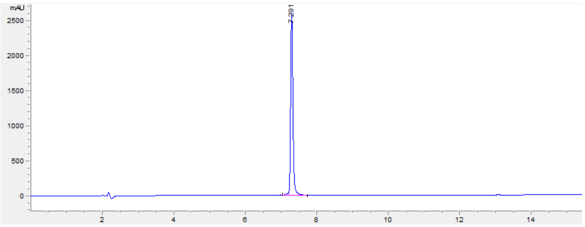

P2-F2C4

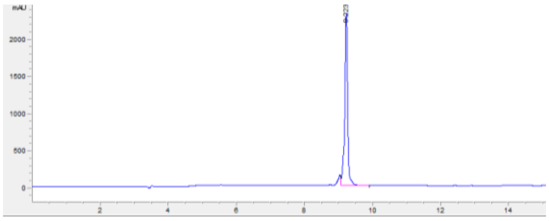

CAM7117

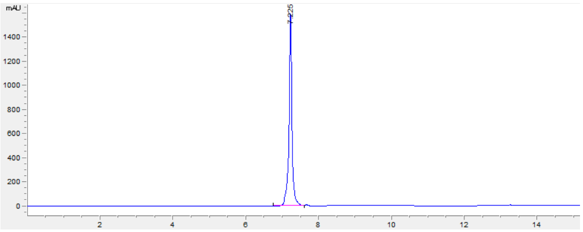

1.3. Computational chemistry experimental

1.3.1. Molecular dynamics

Chains A and D from the crystal structure of human CK2α in complex with a CK2β-derived cyclic peptide called Pc (1-GCRLYGFKIHGCG-13) (PDB: 4IB5)[7] were used as the initial structures for molecular dynamics (MD) simulations. The unresolved CK2α residue Met1 was added using PyMOL[8]. CK2α was capped at its C-terminus by N-methyl while Pc was capped by acetyl and amide groups. Crystallographic water molecules were retained. PDB2PQR[9] was then used to determine the protonation states of residues. Using the LEaP program in the AMBER 14[10] package, each complex was solvated with TIP3P[11] water molecules in a periodic truncated octahedron box such that its walls were at least 10 Å away from the complex, followed by neutralisation of the system with either sodium or chloride ions.

Energy minimisations and MD simulations were carried out by the PMEMD module of AMBER 14, using the ff14SB[12] force field for protein residues and the generalised AMBER force field (GAFF)[13] for the stapled residues. Atomic charges for the stapled residues were derived using the R.E.D. Server,[14] which fits restrained electrostatic potential (RESP) charges[15] to a molecular electrostatic potential (MEP) computed by the Gaussian 09 program[16] at the HF/6-31G* theory level. A total of three independent explicit-solvent MD simulations using different initial atomic velocities were carried out. The SHAKE algorithm[17] was applied to constrain all bonds involving hydrogen atoms, allowing for a time step of 2 fs. A cutoff distance of 9 Å was implemented for nonbonded interactions. The particle mesh Ewald method[18] was used to treat electrostatic interactions with a grid spacing of 1.0 Å and the order of B-spline interpolation set to 4. Energy minimisation was performed for 500 steps with the steepest descent algorithm, followed by another 500 steps with the conjugate gradient algorithm. The system was then heated gradually to 300 K over 50 ps at constant volume before equilibration at a constant pressure of 1 atm for another 50 ps. During the minimisation and equilibration, weak harmonic positional restraints with a force constant of 2.0 kcal mol-1 Å-2 were imposed on the non-hydrogen atoms of the solute. These restraints were removed in a subsequent equilibration run (2 ns) and the production run (100 ns), which were carried out at 300 K and 1 atm. The Langevin thermostat[19] was used to maintain the temperature with a collision frequency of 2 ps-1. Pressure was maintained by a Berendsen barostat[20] with a pressure relaxation time of 2 ps.

1.3.1.1. Ligand-mapping MD

Ten different distributions of chlorobenzenes around apo CK2α were created using Packmol.[21] The LEaP module in the AMBER 14 package was then used to solvate each system with TIP3P water molecules in a periodic truncated octahedron box, such that its walls were at least 10 Å away from the protein, resulting in a final chlorobenzene concentration of ~0.15 M. Minimisation, equilibration and production (20 ns) MD simulations were carried out as described above for the CK2α complexes, for a

cumulative sampling time of 200 ns. The GAFF6 force field was used to describe the chlorobenzenes during the simulations. Atomic charges for chlorobenzene were used as previously described.[22]

The 10 individual runs were combined into a single trajectory for analysis. Chlorobenzene occupancy grids were generated using the cpptraj module of AMBER 14 to bin both carbon and chlorine atoms of chlorobenzenes into 1 Å × 1 Å × 1 Å grid cells. The cutoff isocontour value used for visualisation of chlorobenzene carbon and chlorine atom occupancies is five times the threshold bulk value, which is defined as the highest isovalue at which the respective atoms are detected in the bulk solvent. In order to compare the overlap of the chlorobenzene occupancy maps with known CK2α ligands, the respective ligand-bound CK2α structures were aligned using PyMOL[8] to the average protein structure sampled during the LMMD simulations.

1.3.2. Binding free energy decomposition

The contribution of each Pc peptide residue to the binding free energy of the complex was computed by applying the free energy decomposition method[23] on 200 equally-spaced structures extracted from the last 40 ns of the MD simulations of the CK2α–Pc complex. Binding free energies were calculated in AMBER 14[10] using the molecular mechanics/generalised Born surface area (MM/GBSA) method.[24] The molecular mechanical energies and polar contribution to solvation free energy were computed by the sander module and pbsa program using the modified GB model described by Onufriev *et al*.[25] respectively. The nonpolar contribution to solvation free energy was estimated from the solvent accessible surface area (SASA) using the ICOSA method.[26] A summary of the binding free energy decomposition can be found in **Figure S1**.

1.3.3. Computational alanine scanning

Computational alanine scanning was carried out on 200 equally-spaced structures extracted from the last 40 ns of the MD simulations of the CK2α–Pc complex. The difference in the binding free energy (ΔΔGbind) of the wild type and alanine mutants was calculated using the MM/GBSA method18 with modules in AMBER 14.[10] Molecular mechanical energies were calculated with the sander module. The polar contribution to the solvation free energy was calculated by the pbsa module[27] using the modified GB model described by Onufriev *et al*.[25] while the nonpolar contribution was estimated from the SASA using the linear combinations of pairwise overlaps method,[28] with γ set to 0.0072 kcal mol-1 Å-2 and β to zero.[28] The entropy term was not considered due to the high computational cost and the assumption that the entropy of the mutant does not differ considerably from that of the wild type.[29] The results of the CAS can be found in **Figure S1**.

1.3.4. Binding free energy calculations

Binding free energies for the CK2α complexes were calculated using the molecular mechanics/generalised Born surface area (MM/GBSA) method[24] implemented in AMBER 14.[10] Two

hundred equally-spaced snapshot structures were extracted from the last 40 ns of each of the trajectories, and their molecular mechanical energies calculated with the sander module. The polar contribution to the solvation free energy was calculated by the pbsa[27] module using the modified generalised Born (GB) model described by Onufriev *et al*.[25] while the nonpolar contribution was estimated from the solvent accessible surface area using the molsurf[30] program with γ = 0.0072 kcal Å-2 and β set to zero. Entropies were estimated by normal mode analysis[31] using the nmode program. Due to its computational expense, only 50 equally-spaced snapshots from the last 40 ns of the trajectories were used for analysis.

A summary of the binding free energy calculation can be found in Error! Reference source not found..

1.4. Biophysical experimental

1.4.1. Protein expression and purification

1.4.1.1. CK2α

Three constructs of CK2α were used in this study. For kinase activity assays and competition experiments, CK2α_WT was used (residues 2-329). For ITC CK2α_KA construct was used whilst CK2α_FP10 was used for crystallisation purposes. CK2α_KA (residues 2-329) contained four mutations designed to aid crystallisation by reducing the overall charge of the protein; R21S, K74A, K75A and K76A. CK2α_FP 10 contained one mutation (R21S) and an N-terminal extension GSMDIEFDDDADDDGSGSGSGSGS aimed at mimicking a substrate peptide for CK2α. CK2α_FP10 was cloned into pHAT4 vector and CK2α_KA was cloned into pHAT2 vector to give constructs with cleavable His6-tags. Recombinant plasmids containing one of the three constructs (CK2α_WT/ CK2α_KA/ CK2α_FP10) were introduced into Escherichia coli BL21(DE3) for protein production. Single colonies of the cells were grown in 6 x 1L of 2 x TY with 100 µg/mL ampicillin at 37°C. Isopropylthio-β-D-galactopyranoside (IPTG) was added to a final concentration of 0.4 mM to induce expression when the optical density at 600 nm reached 0.6. The cells were incubated overnight at 25°C then harvested by centrifugation at 4,000 g for 20 minutes. The same extraction and purification procedure were used for all three constructs, with the exception that CK2α_KA used 350 mM NaCl in the buffer, whereas, CK2α_WT and CK2α_FP10 required 500 mM NaCl. The cell pellets were suspended in 20 mM Tris, 350/500 mM NaCl, pH 8.0) and lysed using a high pressure homogeniser. Protease inhibitor cocktail tablets (one tablet per 50 mL extract; Roche Diagnostics) and DNase I were then added. The crude cell extract was then centrifuged at 10,000 g for 45 minutes, the supernatant was filtered with a 0.22 µm filter. The soluble supernatant was applied on a Ni Sepharose Fast Flow6 column at pH 8.0, washed and eluted in 20 mM Tris pH 8.0, 350/500 mM NaCl, 200 mM imidazole. After overnight dialysis into 20 mM Tris, pH 8.0, 350/500 mM NaCl the N-terminal His6-Tag was cleaved overnight by TEV protease and passed through a second metal affinity column to remove uncleaved protein and the protease. The cleaved protein was further purified on a Sepharose Q HP anion-exchange column and the main peak fraction from this column was further purified by gel filtration on a Superdex 75 16/60 HiPrep column equilibrated with Tris 20 mM, pH 8.0, 350/500 mM NaCl. Pure protein was concentrated to 15 mg/mL and flash frozen in liquid N_2.

1.4.1.2. CK2β

pGEX-CK2β construct (1-198) obtained from Victor Bolanos-Garcia (Prof. Tom Blundell's lab) was introduced into *Escherichia coli* BL21(DE3) for protein production. Single colonies of the cells were grown in 6x1 L of 2 x TY with 100 µg/mL ampicillin at 37 °C. Isopropyl thio-β-D-galactopyranoside (IPTG) was added to a final concentration of 0.4 mM to induce expression when the optical density at 600 nm reached 0.6. The cells were incubated overnight at 25 °C then harvested by centrifugation at

4,000 g for 20 minutes. The cell pellets were suspended in 20 mM Tris, 500 mM NaCl, pH 8.5) and lysed using a high-pressure homogeniser.

Protease inhibitor cocktail tablets (one tablet per 50 mL extract; Roche Diagnostics) and DNase I were then added. The crude cell extract was then centrifuged at 10,000 g for 45 minutes, the supernatant was filtered with a 0.22 μm filter. The soluble supernatant was applied on a Glutathione Sepharose column and washed with 5column volumes of loading buffer (20 mM Tris, 500 mM NaCl, pH 8.5) followed by washing with 10 column volumes of cleavage buffer (20 mM Tris 500 mM NaCl, 1mM DTT, 1mM EDTA, pH 8.5). 100μL of the precision protease was loaded onto the column and incubated for 5 hours at 4°C and eluted in the cleavage buffer. The cleaved protein was further purified on a Sepharose Q HP anion-exchange column (gradient 0-500 mM NaCl) and the main peak fraction from this column was further purified by gel filtration on a Superdex 75 16/60 HiPrep column equilibrated with Tris 20 mM, pH 8.5, 500 mM NaCl. Pure protein was concentrated to 15 mg/mL and flash frozen in liquid N_2.

CK2β-RAD display protein used in the BLI experiments has been expressed and purified as reported by Rossmann *et al*.[32]

1.4.2. Fluorescent Polarisation anisotropy

The fluorescent probe was used in the assay was a linear CK2β-based peptide RLYGFKIHPMAYQLQ (CK2β_pep) tat was covalently linked to fluoresceine moiety at N-terminus. The fluorescein-labelled probe was measured using 485 nm excitation and 530 nm emission. Approximate % inhibition of binding of the CK2β-derived peptide to CK2α was determined using a PHERAstar FS plate reader (BMG labtech) using 15 μM and 0 μM of the test compounds at a constant concentration of DMSO. The experiments were performed in a 384 well plate with final concentrations of 450 nM CK2α, 7.4 nM CK2β_pep, 350 mM NaCl, 20 mM Mes pH 6.5. The plates were read after a 30 min incubation period. The experiment was performed in triplicates.

1.4.3. Isothermal Calorimetry Titration

1.4.3.1. Direct binding assay

All ITC experiments were performed at 25°C using a MicroCal ITC-200 (GE Healthcare). CK2α (20 mg/mL, 20 mM tris pH 8.0, 500 mM NaCl) was diluted in Tris buffer (200 mM, NaCl 300 mM, 10% DMSO) and concentrated to 20-50 μM. Compounds in 100× stock solutions were diluted into the same buffer. In a typical experiment CK2α (25 μM) was loaded into the sample cell and 19 injections (2 μL each) with a 2 second duration were performed at 150 second intervals. The syringe was loaded with 200-250 μM ligand and rotated at 750 rpm. Control titrations were performed, and the data fitting was performed with a single site binding model using Origin software.

Binding curves for peptides used in this work can be found in **Table S3**.

1.4.3.2. Competition assay

Experiments were performed at 25°C using a MicroCal ITC-200 (GE Healthcare). CK2α (0.2 mg/mL, 20 mM Tris pH 8.0, 500 mM NaCl) was diluted in Tris buffer (200 mM, NaCl 300 mM, 10% DMSO) and concentrated to 4-6 μM. CK2β (9.3 mg/mL, 20 mM Tris pH 8.5, 500 mM NaCl) was diluted in Tris buffer (200 mM, NaCl, 300 mM, 10% DMSO) and concentrated to 40 μM. In a typical experiment, CK2α was loaded into the sample cell and 19 injections (2 μL each) with a 2 second duration were performed at 150 second intervals. The syringe was loaded with CK2β and rotated at 750 rpm. The same experiment was repeated in the presence of the peptides: compounds at 1 mM in DMSO were added to the solutions of CK2α and CK2β (prepared as described above) to a final concentration of 100 μM. Control titrations were performed, and the data fitting was performed with a single site binding model using Origin software. Binding curves generated during the competition assays can be found in **Table S4**.

1.4.4. BLI assay

Octet RED96 (ForteBio) instrument was used for BLI analysis of biotin-labelled CK2β-RAD/**CAM7117** interactions. Experiments were performed using SA-Streptavidin biosensors (ForteBio), which were regenerated with 10mM glycine, pH 1.7. 20mM TRIS pH 8.0, 350mM NaCl, 0.5% Tween was used as the assay buffer. For binding assays pre-wet sensors were loaded with biotin labelled CK2β-RAD protein at 50 nM in assay buffer. CK2α K74A was prepared at 50 nM in assay buffer and dilution series (from 0 to 10 μM) of **CAM7117** were prepared in the assay buffer. The following experimental setup was used for each measurement cycle: regeneration 3× 5s, baseline 120s, sample loading (CK2β-RAD) 200s, baseline 120s (assay buffer), control loading 200s (RAD), baseline 150s (assay buffer), ligand binding (association) 400s (CK2α K74A and **CAM7117**) and ligand dissociation 600s (assay buffer), followed by regeneration and next cycles. During the control loading stage all remaining streptavidin sites were blocked with biotin labelled RAD that did not include the CK2β loop. Regenerated SA-Streptavidin biosensors loaded with biotin labelled RAD were used to perform referencing. Data was processed using ForteBio Analysis 7.1 software and IC_{50} was calculated using GraphPad. The results are the average of 4 repeats and error shown as SEM.

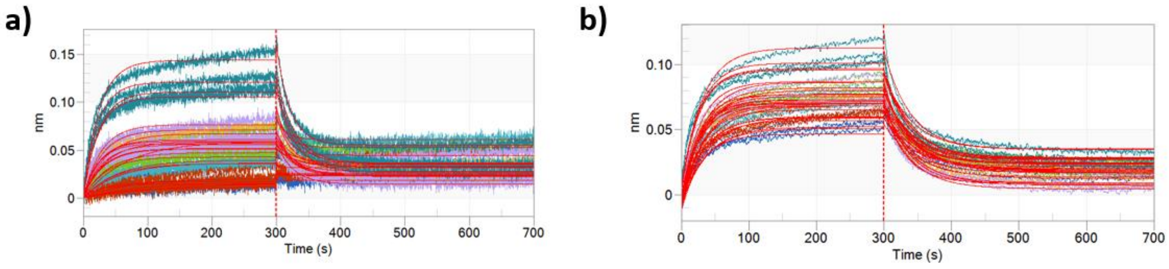

Figure S10- *Sensorgrams of the BLI competition experiments to assess the ability of* ***P2-C4*** *to prevent inhibition of the CK2α/CK2β-RAD complex. a) Concentration of peptide ranging from 10 to 0.025 μM and 0 μM. b) Concentration of peptide ranging from 10 to 5 nM and 0 nM.*

1.4.5. X-ray crystallography

Co-crystals of CK2α and **P1-C4** were generated by screening CK2α_FP10 at 10 mg/mL in 20 mM Tris, pH 8.0, 500 mM NaCl and 500 μM P1-C4 with the JCSG+ screen (molecular dimensions). Drops were set up at 0.2 μL protein solution 0.2 μL screen using the sitting drop vapour-diffusion method. Crystals were observed in a number of conditions in the JCSG+ screen. The condition that yielded the crystals from which the final data set was collected grew in 0.1 M HEPES pH 7.5, 10%(w/v) PEG 8K, 8% v/v Ethylene glycol. The crystals were cryo-cooled in liquid nitrogen in the same solution for data collection.

Crystals of **P2-C4** were generated by using matrix seeding from the co-crystals grown of **P1-C4**. Seeds of CK2α were generated using Micro seed beads (Molecular Dimensions). Co-crystals of CK2α and **P2-C4** were generated by screening CK2α_FP10 at 10 mg/mL in 20 mM Tris, pH 8.0, 500 mM NaCl and 500 μM P2-C4 with the JCSG+ screen (Molecular Dimensions) and the seed stocks obtained from **P1-C4**. Drops were set up at 0.2 μL protein solution + 0.2 μL screen +0.01 μL seed stock using the sitting drop vapour-diffusion method. Crystals were observed in a number of conditions in the JCSG+ screen. The condition that yielded the crystals from which the final data set was collected grew in 0.16 M Calcium Acetate, 0.08 M Sodium Cacodylate, 14.4% PEG 8K, 20% Glycerol.

X-ray diffraction data was collected at Diamond synchrotron radiation sources (beamlines), then processed using the pipedream package by Global Phasing Ltd; structures were solved by using programs from the CCP4 package. [25] Models were iteratively refined and rebuilt by using AutoBuster26 and Coot programs. Ligand coordinates and restraints were generated from their SMILES strings using the Grade27 software package (Global Phasing Ltd).

All coordinates have been deposited to Protein Data Bank and accession numbers and data collection and refinement statistics are shown in. **Table S10**

Table S10 *- Accession numbers, data collection and refinement statistics.*

**PDB**	**6Q38**	**6Q4Q**
**Data Collection**		
Wavelength (Å)	0.9795	0.9795
Resolution range (Å)	63.88-1.74 (1.83-1.74)	66.39-1.445 (1.47-1.45)
Space group	$P\ 2_1\ 2_1\ 2$	$P2_1$
Unit cell (a, b, c, α, β, δ)	60.9 Å, 127.8 Å, 54.5 Å, 90°, 90°, 90°	49.3 Å, 125.2 Å, 66.5 Å, 90.0°, 92.6°, 90.0°
No of total/unique reflections	833029 / 44243	1156827/141193
Multiplicity	18.8 (19.1)	8.2 (7.0)
Completeness (%)	99.7 (99.4)	98.5 (100.0)
Mean I/sigma(I)	11.1 (0.5)	10.6 (0.7)
Wilson B-factor ($Å^2$)	31.44	19.28

R-merge	0.176 (7.965)	0.097 (2.406)
R-meas	0.181 (8.180)	0.103 (2.600)
CC1/2	0.999 (0.419)	0.998 (0.353)
**Refinement**		
Resolution range (Å)	63.88-1.74 (1.78-1.74)	66.39-1.45 (1.49-1.45)
Number of unique/free reflections used	44161/ 3146	139673/10415
Reflections used for R-free	2153 (230)	6925
R-work	0.20 (0.22)	0.21 (0.21)
R-free	0.23 (0.23)	0.22 (0.21)
Number of non-hydrogen atoms	3018	6422
macromolecules	2854	5853
Ligands	54	134
Solvent	110	435
Protein residues	336	670
RMS bonds(Å)	0.015	0.015
RMS angles (°)	1.95	1.59
Ramachandran favored (%)	95.8	97
Ramachandran allowed (%)	4.23	3.02
Ramachandran outliers (%)	0	0
Rotamer outliers (%)	1.9	2.7
Clashscore	4.7	3.4
Average B-factor (Å^2)	56.9	27.6
Macromolecules (Å^2)	56.7	27
Ligands (Å^2)	85.9	36
Solvent (Å^2)	47.4	32.8
Crystallisation conditions	10%(w/v) PEG 8K, 0.1 M HEPES pH 7.5, 8% v/v Ethylene glycol	14.4% PEG 8K, 0.16 M Calcium Acetate, 0.08 M Sodium Cacodylate pH 6.5, 20% Glycerol

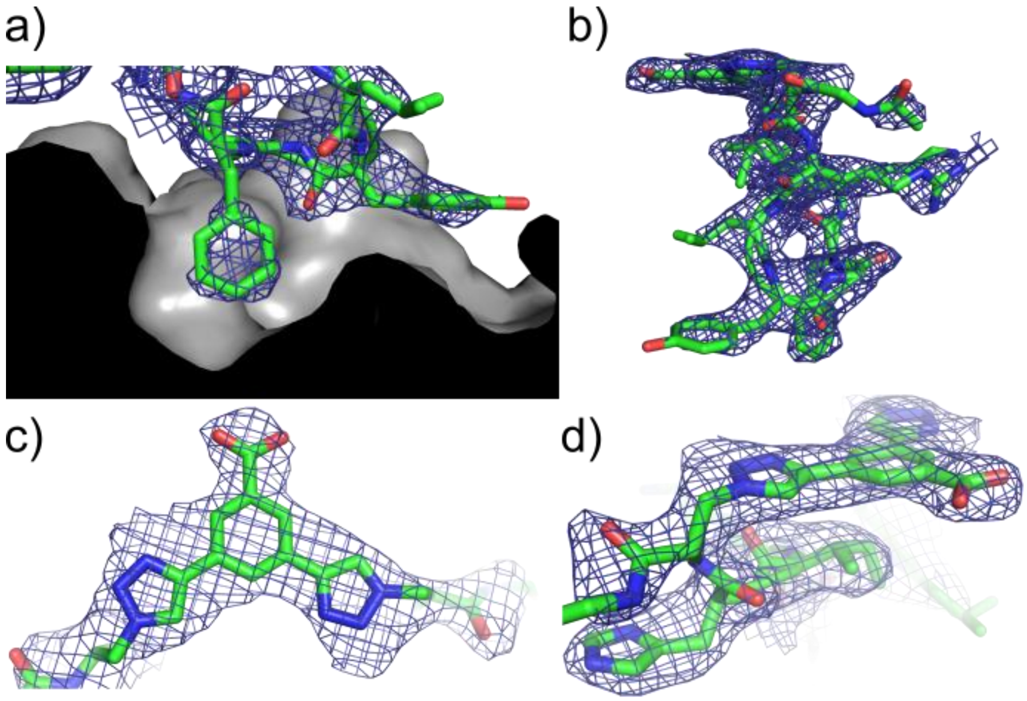

Figure S11- Electron density from the co crystal structure of ***P1-C4*** bound to CK2α. All maps are the 2Fo-Fc contoured at 1σ a) Cross section showing the electron density of ***P1-C4*** bound in the Phe and Tyr b) The electron density for the whole peptide. c) The electron density for the linker ***C4***. d) The electron density showing the linker ***C4*** interacting with the Ile9.

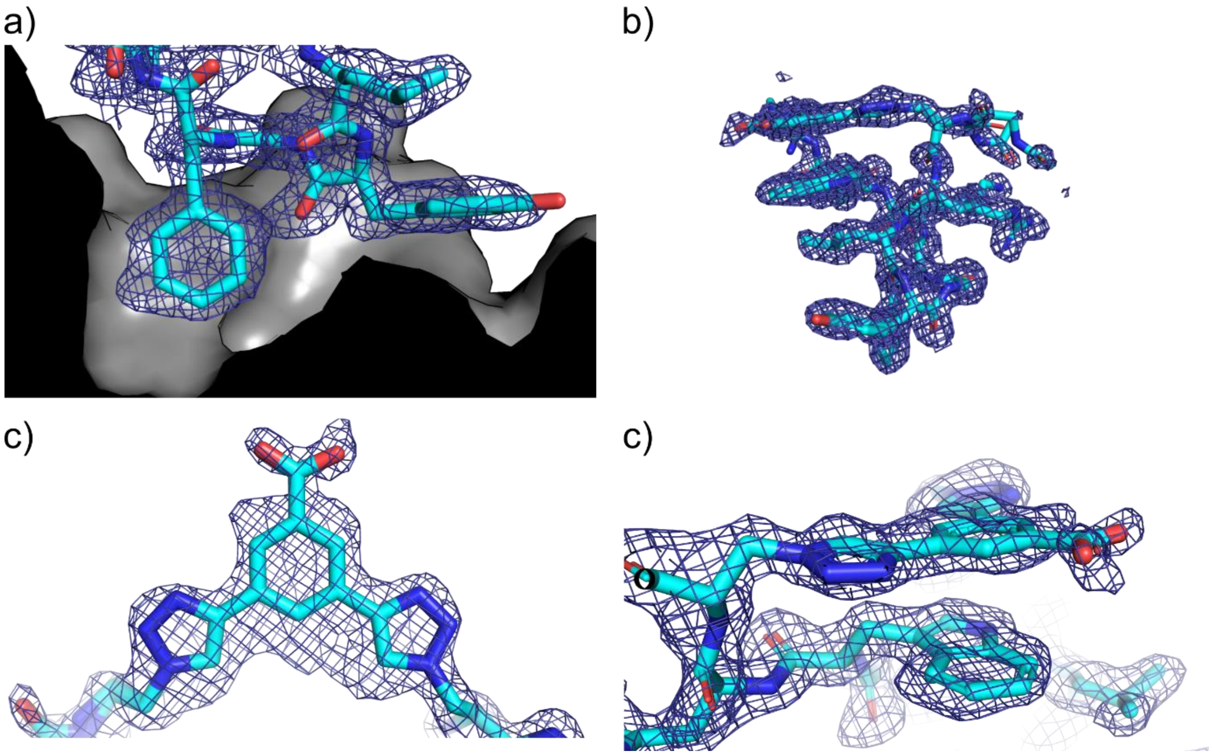

Figure S12 - Electron density from the co crystal structure of ***P2-C4*** bound to CK2α. All maps are the 2Fo-Fc contoured at 1σ a) Cross section showing the electron density of ***P2-C4*** bound in the Phe and Tyr b) The electron density for the whole peptide. c) The electron density for the linker ***C4***. d) The electron density showing the linker ***C4*** interacting with the Trp9.

1.4.6. Kinase assay

The kinase assays were performed using the ADP-Glo™ kinase assay kit (Promega). 50 nM CK2α_WT was incubated in the kinase reaction buffer (40 mM Tris pH 7.5, 200 mM NaCl, 20 mM $MgCl_2$, 0.1 mg/mL BSA, 25 μM ATP, 50 μM substrate peptide (RRRADDSDDDD, Enzo Life Sciences Inc. or eIF2β peptide MSGDEMIFDPTMSKKKKKKKKP), 5% (v/v) DMSO) in the presence of different concentrations of the inhibitor at 25 °C for 120 min. When eIF2β peptide was used 50 nM CK2β_WT was added to the solution containing 50 nM of CK2α_WT to pre-form the heterodimer. 5 μL aliquots of the kinase reaction were quenched with 5 μL of ADP-glo™ solution. After 45 min 10 μL of the kinase detection reagent was added and maintained at 25 °C for 20 minutes. The luminescence was recorded using a PHERAstar FS plate reader (BMG LABTECH) with an integration time of 1 sec. Percentage inhibition was calculated relative to a DMSO control after subtraction of the measurement for basal ATPase activity in the absence of substrate. The results are the average of at least three independent repeats and are shown in **Figure S2**.

1.5. Biological experimental

1.5.1. Serum stability test

500 µL of PBS buffer supplemented with 20% (v/v) of human serum was allocated into an Eppendorf tube and temperature kept at 37 °C for 15 minutes before commencing the experiment. 5 µL of the peptide from 10 mM stock solution in DMSO was added. Caffeine was added as an internal standard (10 µL of a 15 mg/mL solution in MQ water). At specific intervals, 50 µL of the reaction mixture was taken and quenched with 100 µL of a 1:1 mixture of 96% Ethanol:DMSO. The suspension was spun at 13400 *g* for 10 minutes. 100 µL of the supernatant was analyzed using C-18 HPLC with an eluting gradient 5-95% ACN (0.05% TFA) in water (0.05% TFA) over 15 minutes (90 µL injection volume). Percentage of intact starting peptide was monitored over 24 h (calculated as the ratio of the area of the peak corresponding to the intact peptide to the area of the peak of caffeine). The experiment was performed in duplicates.

1.5.2. Tissue culture

All cell lines used were supplied by AstraZeneca cell bank as mycoplasma free. U2OS bone osteosarcoma and HCT116 colon carcinoma cells were maintained in RPMI-1640 (1x, Sigma Aldrich, R0883) supplemented with 2 mM L-glutamine (Gibco, 25030-149) and fetal bovine serum (FBS, Gibco Life Technologies, 10270-106) at a final concentration of 10%, hereafter known as growth medium. All cells were grown at 37°C/5% CO_2 in a humidified environment and all the assays were performed using these culturing conditions.

1.5.3. Proliferation assay

HCT116 cells were seeded at 2000 cells per well into two flat-bottomed 384-well plates (PerkinElmer CellCarrier Ultra) in 40 µL of growth medium. After 24 hours, the peptides dissolved in DMSO (10 mM) were added to the cells (to final concentrations of 100, 75, 50, 32, 25, 16, 10, 7.7, 5, 3.2, 0 µM) using HP Tecan dispenser and DMSO normalised to a final concentration of 1% (v/v). Cells were then incubated in the presence of the compounds for 4 days and the rate of growth monitored with IncuCyte Zoom (plate *day 4*). To one plate (*day 0*), 5 µL of Saponin (0.25% w/v in 5 mM EDTA in TBS buffer) and 10 µL of SYTOX™ green reagent (2 µM in TBS buffer containing 5 mM EDTA) were added after dosing. The plate was left in the dark at rt for 4 hours before imaging with Thermo Scientific™ CellInsight™ CX5 High Content Screening (HCS) Platform using 10X magnification. The same procedure was repeated for plate *day 4* after 4 days of incubation. Percentage of growth inhibition was calculated relative to DMSO controls, normalised against day 0 and GI_{50} values were calculated using Graphpad Prism. The results are the average of at least four independent repeats.

1.5.4. Viability assay

HCT116 cells were seeded at 5000 cells per well into a flat-bottomed 384-well plate (PerkinElmer CellCarrier Ultra) in 40 μL of growth medium. After 24 hours, the compounds (10 mM stock in DMSO) were added to the cells to final concentrations of 100, 75, 50, 32, 25, 16, 10, 7.7, 5, 3.2 and 0 μM using HP Tecan dispenser and DMSO normalised to a final concentration of 1% (v/v). After 4 hours, 20 μL of CytoTox-Glo™ Cytotoxicity Assay Reagent (Promega) were added to all well. After 15 minutes at room temperature, luminescence was read on a multimode plate reader (EnVision, PerkinElmer). 40 μL of Lysis Reagent (prepared using 33 μL Digitonin in 5 mL of assay buffer) were added to all wells, mixed, and incubated at room temperature for 15 minutes. Luminescence of total dead cells was measured. The luminescent contribution of previously viable cells (after lysis) was calculated by subtracting the luminescent signal resulting from experimental cell death from total luminescence death. The results are the average of at least three independent repeats.

1.5.5. Co-localisation experiments

Confocal imaging of fixed U2OS cells was conducted using a Yokogawa Cell Voyager CV8000 confocal microscope with a 40X water immersion objective. Images were acquired with excitation at 488, 561 and 405 nm and emissions detected with a 525/50, 600/37, 445/60 nm BP filter for the green, red and blue channel respectively.

The following primary antibodies were used:

Antibody	Organelle stained	Supplier and catalogue number	Working concentration
EEA1	Early endosomes	Abcam, ab2900	1:4000
Rab7	Late endosomes	Abcam, ab50533	1:4000
LAMP1	Lysosomes	Cell Signalling, 9091	1:4000
TGN46	Trans Golgi	Abcam, ab50595	1:750
ZFPL1	Cis Golgi	Sigma, HPA014909	1:500
Calnexin	Endoplasmic reticulum	Invitrogen, PA5-34665	1:750
CK2β	CK2β subunit of CK2	Fitzgerald, 20R-2974	1:2000

Secondary antibodies (goat anti-rabbit IgG (H+L) AlexaFluor 568 and goat anti-mouse IgG (H+L) AlexaFluor 568) were used at a working concentration of 1:500. Hoechst nuclei stain was used to a working concentration of 1:5000. FITC-labelled peptides were used at 1.25 μM final concentration unless otherwise stated.

U2OS cells were seeded at 2500 cell per well into a flat-bottomed tissue 384-well plate (PerkinElmer CellCarrier Ultra) in a volume of 40 μL of growth medium and cultured for 24 h before commencing the experiment. After 24 hours, FITC-labelled compounds dissolved in DMSO (1 mM) were added to the cells using HP Tecan dispenser and DMSO normalised to a final concentration of 0.3% (v/v). Cells were fixed after 360, 180, 120, 60, 30, 15, and 5-minute incubation using 40 μL of 8% PFA (final PFA

concentration 4%). Cells were fixed for 15 minutes, washed with PBS (3x) and blocked with 3% BSA + 0.1 % Triton X-1000 for 5 minutes. The blocking solution was then removed and 40 µL of the primary antibodies in BSA added. The plate was stored in the dark, at 4 °C overnight. The primary antibodies were washed with PBS (3x) and 40 µL of the secondary antibodies and Hoechst nuclei stain in BSA added. The plate was kept at 4 °C for 1 hour, washed with PBS (3x) and imaged as described above. Images were analysed using Fiji (ImageJ) software.

1.6. References

1. Tobe, Y. *et al.* m-Diethynylbenzene Macrocycles: Syntheses and Self-Association Behavior in Solution. *J. Am. Chem. Soc.* **124,** 5350-5364 (2002).
2. Pal, R., Clark, R. J., Manoharan, M. & Alabugin, I. V. Fast oxy-cope rearrangements of bis-alkynes: Competition with central C-C bond fragmentation and incorporation in tunable cascades diverging from a common bis-allenic intermediate. *J. Org. Chem.* **75,** 8689-8692 (2010).
3. Fischer, N. *et al.* Sensitivities of some imidazole-1-sulfonyl azide salts. *J. Org. Chem.* **77,** 1760–1764 (2012).
4. Zhang, L., Kauffman, G. S., Pesti, J. A. & Yin, J. Rearrangement of N_{α} -Protected -L -Asparagines with Iodosobenzene Diacetate. A Practical Route to β-Amino- L -alanine Derivatives. *J. Org. Chem.* **62,** 6918-6920 (1997).
5. Förster, B., Bertran, J., Teixidor, F. & Viñas, C. Synthesis of new arylcarboranes as precursors for oligomers. *J. Organomet. Chem.* **587,** 67-73 (1999).
6. Lau, Y. H., Wu, Y., de Andrade, P., Galloway, W. R. J. D. & Spring, D. R. A two-component ‘double-click’ approach to peptide stapling. *Nat. Protoc.* **10,** 585–594 (2015).
7. Raaf, J. *et al.* First structure of protein kinase CK2 catalytic subunit with an effective CK2??-competitive ligand. *ACS Chem. Biol.* **8,** 901-907 (2013).
8. De Lano, W. L. *The PyMOL Molecular Graphics System, DeLano Scientific, San Carlos, CA, USA.* (2002).
9. Dolinsky, T. J., Nielsen, J. E., McCammon, J. A. & Baker, N. A. PDB2PQR: An automated pipeline for the setup of Poisson-Boltzmann electrostatics calculations. *Nucleic Acids Res.* **32,** W665-667 (2004).
10. Case, D. A., *et al*. *P. A. (2014) AMBER 14, University of California, San Francisco*
11. Jorgensen, W. L., Chandrasekhar, J., Madura, J. D., Impey, R. W. & Klein, M. L. Comparison of simple potential functions for simulating liquid water. *J. Chem. Phys.* **79,** (1983).
12. Maier, J. A. *et al.* ff14SB: Improving the Accuracy of Protein Side Chain and Backbone Parameters from ff99SB. *J. Chem. Theory Comput.* **11,** 3696-3713 (2015).
13. Wang, J., Wolf, R. M., Caldwell, J. W., Kollman, P. A. & Case, D. A. Development and testing of a general Amber force field. *J. Comput. Chem.* **25,** 1157-1174 (2004).
14. Vanquelef, E. *et al.* R.E.D. Server: A web service for deriving RESP and ESP charges and building force field libraries for new molecules and molecular fragments. *Nucleic Acids Res.* **39,** W511-W517 (2011).
15. Cornell, W. D., Cieplak, P., Bayly, C. I. & Kollman, P. A. Application of RESP Charges To Calculate Conformational Energies, Hydrogen Bond Energies, and Free Energies of Solvation. *J. Am. Chem. Soc.* **115**, 9620-9631 (1993).
16. Frisch, M. J., *et al*. *Gaussian 09, Revision B.1, Gaussian, Inc., Wallingford CT* (2009).
17. Ryckaert, J. P., Ciccotti, G. & Berendsen, H. J. C. Numerical integration of the cartesian equations of motion of a system with constraints: molecular dynamics of n-alkanes. *J. Comput. Phys.* **23,** 327-341 (1977).
18. Darden, T., York, D. & Pedersen, L. Particle mesh Ewald: An N·log(N) method for Ewald sums in large systems. *J. Chem. Phys.* **98,** 10089(1993).
19. Lzaguirre, J. A., Catarello, D. P., Wozniak, J. M. & Skeel, R. D. Langevin stabilization of molecular dynamics. *J. Chem. Phys.* **114,** 2090 (2001).

20. Berendsen, H. J. C., Postma, J. P. M., Van Gunsteren, W. F., Dinola, A. & Haak, J. R. Molecular dynamics with coupling to an external bath. *J. Chem. Phys.* **81,** 3684 (1984).

21. Martinez, L., Andrade, R., Birgin, E. G. & Martínez, J. M. PACKMOL: A package for building initial configurations for molecular dynamics simulations. *J. Comput. Chem.* **30,** 2157, 2164 (2009).

22. Tan, Y. S., Spring, D. R., Abell, C. & Verma, C. The use of chlorobenzene as a probe molecule in molecular dynamics simulations. *J. Chem. Inf. Model.* **54,** 1821-1827 (2014).

23. Gohlke, H., Kiel, C. & Case, D. A. Insights into protein-protein binding by binding free energy calculation and free energy decomposition for the Ras-Raf and Ras-RalGDS complexes. *J. Mol. Biol.* **330,** 891-913 (2003).

24. Srinivasan, J., Cheatham, T. E., Cieplak, P., Kollman, P. A. & Case, D. A. Continuum solvent studies of the stability of DNA, RNA, and phosphoramidate-DNA helices. *J. Am. Chem. Soc.* **120**, 9401-9409 (1998).

25. Onufriev, A., Bashford, D. & Case, D. A. Exploring Protein Native States and Large-Scale Conformational Changes with a Modified Generalized Born Model. *Proteins Struct. Funct. Genet.* **55,** 383-394 (2004).

26. Rarey, M., Kramer, B., Lengauer, T. & Klebe, G. A fast flexible docking method using an incremental construction algorithm. *J. Mol. Biol.* **261,** 470-489 (1996).

27. Luo, R., David, L. & Gilson, M. K. Accelerated Poisson-Boltzmann calculations for static and dynamic systems. *J. Comput. Chem.* **23,** 1244-1253 (2002).

28. Weiser, J., Shenkin, P. S. & Still, W. C. Approximate atomic surfaces from linear combinations of pairwise overlaps (LCPO). *J. Comput. Chem.* **20,** 217-230 (1999).

29. Massova, I. & Kollman, P. A. Computational alanine scanning to probe protein-protein interactions: A novel approach to evaluate binding free energies. *J. Am. Chem. Soc.* **121,** 8133–8143 (1999).

30. Connolly, M. L. Analytical molecular surface calculation. *J. Appl. Crystallogr.* **16,** 548-558 (1983).

31. Brooks, B. R., Janežič, D. & Karplus, M. Harmonic analysis of large systems. I. Methodology. *J. Comput. Chem.* **16,** 1522-1542 (1995).

32. Rossmann, M., Greive S. J., Moschetti T., Dinan T., Hyvönen M., Development of a multipurpose scaffold for the display of peptide loops, *Protein Engineering, Design & Selection*, **6**, 419–430 (2017).
